# Ultrahigh-radiance TTA-based OLED with 13 kA cm^−^^2^ current injection

**DOI:** 10.1038/s41377-025-02134-z

**Published:** 2026-01-27

**Authors:** Jichen Zhao, Yu Mao, Wansheng Liu, Zengyi Peng, Xu Wang, Jianhua Zou, Jianbin Wang, Dan Chen, Dongge Ma, Hongbin Wu, Bin Hu, Junbiao Peng

**Affiliations:** 1 National Key Laboratory of Luminescence Materials and Devices, South China University of Technology, Guangzhou, China; 2 Jihua Laboratory, Foshan, Guangdong Province China; 3 Guangdong Basic Research Center of Excellence for Energy and Information Polymer Materials, Guangzhou, China; 4 Spin-X Institute, South China University of Technology, Guangzhou, China

**Keywords:** Organic LEDs, Polymers

## Abstract

Organic semiconductors have been widely utilized in displays, solar cells, detectors, and other fields due to their tunable optoelectronic properties and simple fabrication processes. However, fabricating organic electrically pumped lasers remains an unresolved challenge. The low mobility of organic molecules struggles to sustain the current injection required for electrically pumped lasing, and the free carriers and triplets generated under high current density also quench the gain characteristics. In device fabrication, high-conductivity electrodes and resonant cavities are inevitably accompanied by optical losses, which decrease the quality factor of the resonator and further elevate the threshold current density for electrical pumping. Here, we fabricated an organic light-emitting diode (OLED) with triplet-triplet annihilation (TTA) characteristics and excellent electrical performance, capable of injecting a current density of 13 kA cm^−^^2^ under 15-ns electrical pulse driving. By leveraging short-pulse driving to mitigate triplet accumulation and utilizing the TTA effect to suppress singlet-triplet annihilation (STA), the device can still remain nearly 1% external quantum efficiency (EQE) with 1 kA cm^−^^2^ current injection and achieved a record-high output power of 56 W cm^−^^2^, which can sustain population inversion. The OLED was integrated into a high-quality distributed Bragg reflector (DBR) microcavity with ultrathin electrodes, realizing narrow-band light emission with a spectral linewidth of 5.5 nm under 13 kA cm^−^^2^ current injection. This work paves the way for future fabrication of organic electrically pumped lasers with gain characteristics.

## Introduction

Organic semiconductor materials, which possess conjugated molecular structures and the ease of processing, are widely used in optoelectronic devices^1–4^. Their luminescent properties can be tuned through molecular design and exhibit high gain characteristics, which establishes them as promising laser media^5–9^. Planar microcavities offer facile fabrication and effective coupling to organic gain layers. Organic optically pumped microcavity lasers are well-established, exhibiting thresholds <1 μJ cm^−2^, linewidths <1 nm, and wavelength-tunable emission via cavity length modulation^8,10–14^. However, there are still many difficulties in realizing electrically pumped organic microcavity laser devices. Limited by the low mobility of organic semiconductor materials, it is challenging for organic optoelectronic devices to achieve high current density (kA cm^−2^) injection and realize a population inversion. At present, through heat management such as electrical pulse driving with a short duration shortened to nanoseconds and light-emitting areas are reduced to the micrometer level, organic optoelectronic devices can achieve high current density injection^7,15–18^. This places extremely high demands on the resistance and capacitance characteristics of the fabricated device^19,20^. The indium tin oxide (ITO) and metal electrodes in organic optoelectronic devices exhibit strong optical absorption within the high-reflectivity band of reflector^15,17,21–24^. Incorporating these electrodes into microcavities degrades the cavity quality factor, leading to both elevated lasing thresholds and broadened emission linewidths. This inherent conflict between current injection and optical confinement capability fundamentally limits the development of electrically pumped organic laser devices^25–28^. Furthermore, under high current injection, organic materials form a large number of harmful triplet excitons that quench singlet luminescence, known as the STA, which significantly increases the threshold of stimulated emission for organic laser materials and limits the realization of electrically pumped organic laser devices^21,29^. The use of thermally activated delayed fluorescence (TADF) characteristics or triplet up-conversion technology can reduce the negative impact of triplets, which has been proven in OLEDs^30–33^. However, it remains unknown whether they still work under high current density injection.

In this work, we fabricated a 20 nm-thick ITO substrate with excellent electrical properties and minimal optical loss, which was integrated into a microcavity. By employing 15-ns short pulses to drive the OLED, we avoided triplet accumulation while leveraging an emitter layer with TTA characteristics to mitigate STA effects. The resulting top-emitting OLED sustained a record-high current density of 13 kA cm^−2^ and achieved a breakthrough output power of 56 W cm^−2^, theoretically demonstrating population inversion. Within the high-quality-factor DBR microcavity architecture where electrode-induced optical losses were overcome, we attained electroluminescence (EL) with a 5.5 nm full width at half maximum (FWHM) in 13 kA cm^−2^ current injection, this can be comparable to the emission linewidth of an optically pumped microcavity without electrode below threshold. Validating the structure’s potential for realizing organic electrically pumped lasers.

## Results

### Narrow-spectral-bandwidth microcavity OLED with high current injecting

**Fig. 1:**
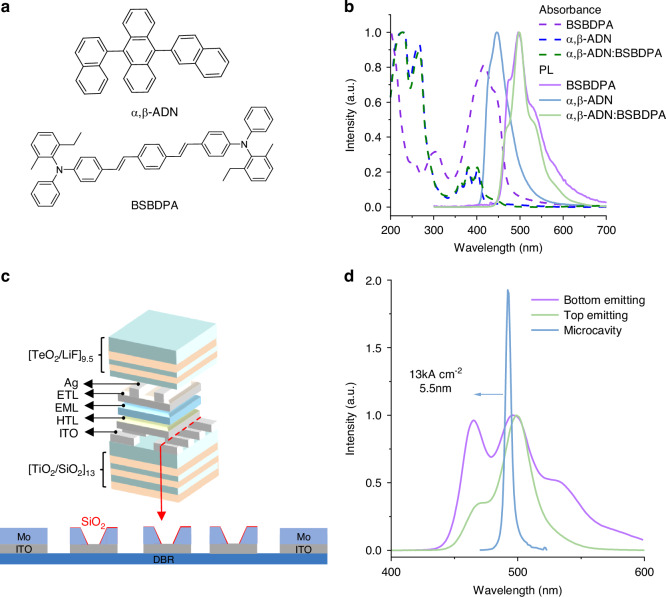
Basic spectrum for α,β-ADN: BSBDPA-based film and OLED. **a** Chemical structure of α,β-ADN and BSBDPA molecules. **b** Absorption and PL spectrum of 120 nm BSBDPA, α,β-ADN and α,β-ADN: BSBDPA(5%wt) films. **c** Microcavity OLED device structure. **d** EL spectrum of BE, TE, and microcavity OLEDs. The active area is 10^−^^4^ cm^−^^2^, driven by 15 ns-10 Hz pulsed voltage

The ADN series molecules serve as conventional TTA host materials, with α,β-ADN demonstrating superior morphological stability^34^. We therefore selected α,β-ADN as the host matrix for fluorescent dopant integration. The chemical structure of α,β-ADN and fluorescent dopant (BSBDPA) is shown in Fig. 1a. The absorption spectrum of BSBDPA overlaps with the emission of α, β-ADN shown in Fig. 1b, inducing the efficient Förster energy transfer (FRET) of the singlets from α,β-ADN to BSBDPA. Due to the severe concentration quenching, we chose α,β-ADN: BSBDPA (5%wt) blend films, which possess the highest photoluminescence quantum yields (PLQYs) of 62% as the emitting layer to fabricate the OLED (Fig. S2a). The OLED structure of the device is shown in Fig. 1c. The device fabrication initiated with the deposition of a multilayer dielectric stack called DBR on sapphire substrates to serve as the bottom reflector. Subsequently, an ITO/Mo/SiO₂ composite structure was deposited as the bottom electrode. The 20 nm ultrathin ITO anode achieved ultralow optical absorption. Low-resistance Mo deposited on the ITO reduced the series resistance. A SiO₂ pixel defined layer (PDL) patterned μm-scale emission zones between the ITO and Mo electrodes. Organic functional layers and metal electrodes were then sequentially deposited via thermal evaporation. Finally, a multilayer dielectric stack was deposited as the top reflector to complete the device structure. The energy level diagrams of bottom-emitting and top-emitting devices are shown in Fig. S1. MeO-TPD: F4-TCNQ (4 wt%) composite demonstrates enhanced hole-transport properties, serving as the hole-injection layer (HIL)^16^. Under high-current-density operation (>1 kA cm^−2^), charge accumulation at heterojunction interfaces quenches singlet excitons via the singlet-polaron annihilation (SPA) effect, leading to pronounced efficiency roll-off in OLEDs^35^. So we selected MeO-TPD as the hole-transport layer (HTL) to minimize interfacial energy barriers. To achieve charge balance, we incorporated Bebq_2_:Liq (40 wt%) as the electron transport layer (ETL). Liq additives optimize the work function of Bebq_2_ for energy-level matching with Mg:Ag (1:9) cathodes. Ultimately, this device can withstand a current density injection of 13 kA cm^−^^2^, and its bottom-emission (BE), top-emission (TE), and top-emission spectra with a DBR microcavity are shown in Fig. 1d. It presents ultra-high output power of 56 W cm^−^^2^ in the TE device and ultra-narrow linewidth spectrum of 5.5 nm in the DBR-DBR microcavity device.

### Optical gain characterization of film

The records of the PLQY and the decay lifetime of the α,β-ADN doped film and CBP film are 62%, 87% and 1.24 ns, 1.37 ns, respectively, exhibiting that BSBDPA possesses a high radiative rate. The detailed photophysical data of the doped films at different doping concentrations are shown in Fig. S2. As is well known, a higher radiation rate is related to a larger stimulated emission cross section.

**Fig. 2:**
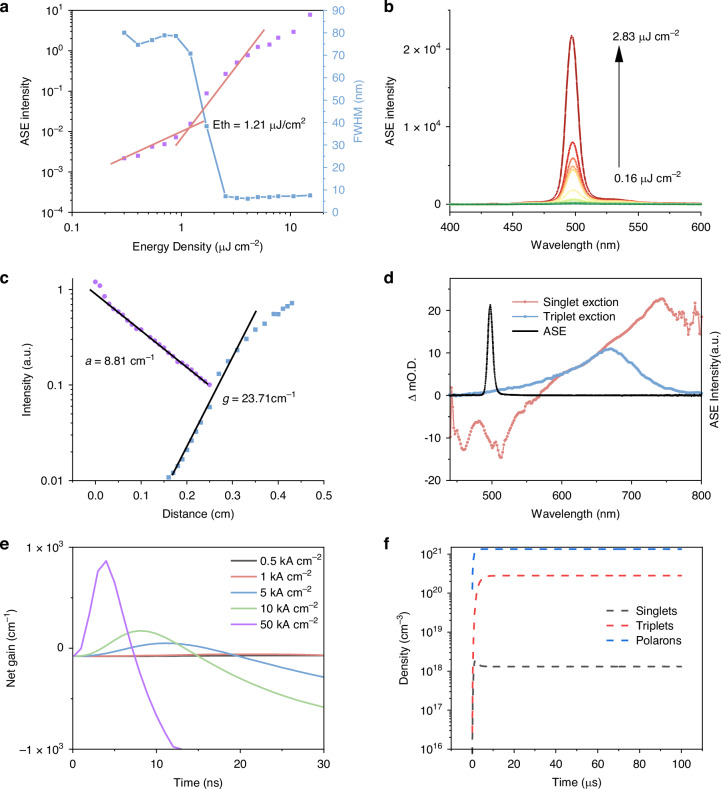
Optical gain characterization for film and gain simulation for OLEDs. **a** Intensity and FWHM of 120 nm α,β-ADN:BSBDPA(5%wt) film at different excitation fluences of a pulsed laser (duration, 230 fs; wavelength, 343 nm; repetition rate, 18 Hz; beam size, 1.0 mm × 4.3 mm) **b** Edge emission PL spectrum of α,β-ADN:BSBDPA(5%wt) film. **c** Edge emission PL intensity versus distance (extract the gain coefficient g and the loss coefficient a). **d** Femtosecond transient absorption spectroscopy(fs-TAS) and ASE spectrum of 120 nm α,β-ADN: BSBDPA(5%wt) film, triplet absorption spectrum of BSBDPA was measured in oxygen-free chlorobenzene solution. **e** Simulation of net gain versus time of OLEDs at different current density. **f** Simulation of singlets triplets and polarons density versus time in the OLEDs at 4 kA cm^−2^(net gain=0)

**Fig. 3:**
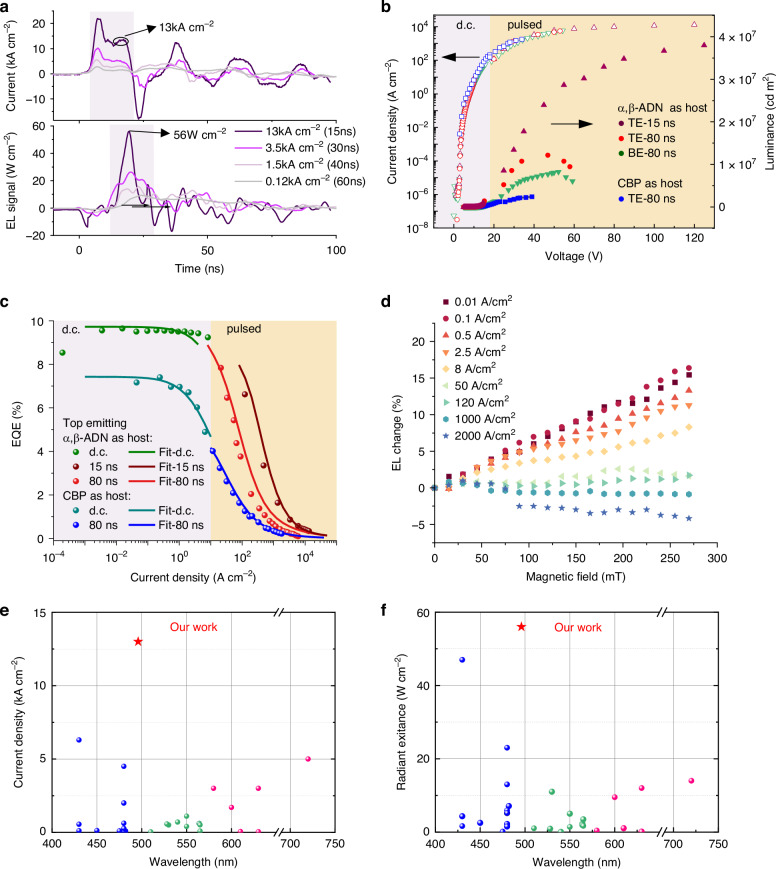
Characterization of the ultrahigh-radiance OLED. **a** Transient current and EL signal of the TE OLEDs with different pulse widths. **b** Current density-Voltage-Luminance(I-V-L) characteristics of the α,β-ADN:BSBDPA(5%wt) and CBP:BSBDPA(5%wt) based OLEDs. **c** Simulated and experimental EQE roll-off characteristics of the α,β-ADN: BSBDPA (5%wt) and CBP: BSBDPA (5%wt) based OLEDs. **d** Magnetic field induced EL changes versus magnetic field intensity for α,β-ADN: BSBDPA(5%wt) based OLED. **e**, **f** Literature-compiled^15^ and newest^40^ OLED statistical: Maximum radiant exitance at different emission wavelengths within different current injection. Our work presents the highest radiant exitance of the 56 W cm^−^^2^ and highest current injection of 13 kA cm^−^^2^

Amplified spontaneous emission (ASE) measurements were performed on both α,β-ADN and CBP doped solid state films to investigate the gain performance of BSBDPA. The host-guest energy transfer doping system was constructed to avoid the concentration quenching effect, reduce the self-absorption loss, and lower the threshold. The ASE threshold is determined by the non-linear change point of the slope of the pump fluence versus output emission intensity. According to Fig. S2, the ASE threshold of the doped film first decreases and then increases with the doping concentration under the combined effect of the Forster energy transfer rate and the concentration quenching effect. At low doping percentage, the Forster energy transfer is not sufficient, as the doping percentage increases, the concentration quenching effect increases, both leading to the threshold increase. We get the optimum doping percentage of 5%, Fig. 2a shows an ASE threshold as low as 1.21 μJ cm^−^^2^ in the α,β-ADN: BSBDPA (5 wt%) doped film, the FWHM of the emission spectra decreases from 90.0 to 11.0 nm with increasing pump fluence as shown in Fig. 2b. The net gain and loss coefficients were estimated by the variable stripe length (VSL) method, which are 23.71 and 8.81 cm^−1^, respectively, as shown in Fig. 2c. Figure 2d shows triplet absorption spectrum of BSBDPA which was measured in oxygen-free chlorobenzene solution. The negligible overlap between the triplet absorption spectrum and the ASE spectrum indicated the material’s extremely low triplet absorption loss. Through fitting the 670 nm dynamic process, the triplet lifetime of BSBDPA was determined to be 12.64 μs (Note Fig. 3b).

### Gain simulation of OLEDs

Based on these low-threshold and low-loss systems, we will first explore the possibility of achieving electrically pumped lasing through kinetic simulation before fabricating the OLED device. The extraction of relevant parameters and the simulation process are presented in the Supplementary Note 1. As we mentioned above, there are several decay processes of singlets in organic systems, including fluorescence emission, intersystem crossing (ISC), STA, and SPA. After extracting the rate constants, combined with the numerical solution of the dynamic equations, we could quantitatively characterize the exciton or carrier concentration versus time and current density, and then we can simulate the rate of each decay channel under different current densities to find out the most dominant decay channel under electrical injection.

According to the simulation results, as shown in Fig. S3, the following features can be found: First, the ISC and SPA decay process are much smaller than the fluorescence emission and STA decay process; Second, with the increase of current density, the rise time of fluorescence emission and STA curves becomes faster, and the corresponding peak time is earlier; Third, at a small current density, fluorescence emission is the most dominant decay channel, and with the increase of current density, the proportion of STA increases, which is the most dominant one. The STA ratio increases and eventually becomes much larger than the fluorescence emission. This suggests that STA is the dominant decay process at high current densities.

The simulated curve of the gain characteristics of the device under electrical injection is shown in Fig. 2e. We can see that the maximum net gain coefficient *g* is initially negative and then increases as the current density (*J*) increases. In particular, for α,β-ADN doped devices, *g*~ 0 when *J*_*th*_ ~ 4 kA cm^−^^2^, which corresponds to a singlet density of 1.1 × 10^18^ cm^−^^3^(Fig. 2f), is larger than the singlet density corresponding to the ASE threshold under optical pumping which is 2.0 × 10^17 ^cm^−^^3^, due to the singlets annihilation under current injection, especially the STA mentioned above. The curves of the net gain coefficient versus time at different current densities show that when *J* < threshold, the curve is always negative, and when *J* > threshold, the curve first rises and then falls with time, which is due to the fact that exciton generation takes some time, and on the other hand, the continuous electrical injection accumulates more and more triplets, causing a strong quenching of the singlets. Besides, the time window (*T*) of *g*> 0 first increases and then decreases as the current density increases, and there is an extreme value of 10.5 ns. Therefore, according to our simulation, direct electrically pumped organic lasers are most likely to be in the form of ns-pulses. Moreover, the pulse width and current density of the electrical pulse are related to the gain time window and must be adjusted for specific materials; otherwise, the laser may be quenched.

**Fig. 4:**
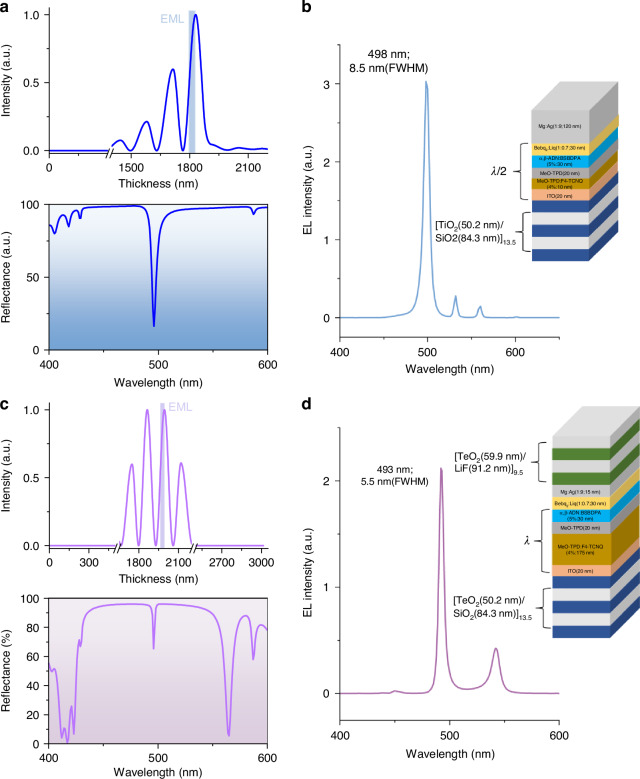
Characterization of the microcavity OLED. **a** Optical simulation of DBR-MgAg microcavity: Distribution of transverse electric field at wavelength of 496 nm within the device (above); Reflection spectrum on the MgAg side of the device(below). The simulation process is shown in the supplementary note. 2. **b** EL spectrum on the MgAg side of the device with 13 kA cm^−^^2^ current injection. **c** Optical simulation of DBR microcavity: Distribution of transverse electric field at wavelength of 496 nm within device(above); Reflection spectrum on the top DBR side of the device(below). **d** EL spectrum on the top DBR side of the device with 13 kA cm^−2^ current injection

The gain performance of the CBP-doped devices is much worse than that of the α, β-ADN-doped device, as shown in Note Fig. 4. The threshold also reaches an almost impossible current density of 23 kA cm^−2^. This may be attributed to the higher k_ST_. So only the α, β-ADN-doped devices are discussed parametrically to find the factors that favor laser realization. With all other parameters held constant, the effects of the STA rate and the TTA rate on the gain profile are considered separately. As shown in Fig. S4, as STA rate increases, $g$ decreases significantly, and the threshold current density corresponding to *g* = 0 increases dramatically. Increasing the TTA rate increases $g$ and decreases the threshold current density, and there we can see that increasing the TTA rate favors the widening of the gain time window for *g*> 0. As shown in the net gain coefficient-time curve of the device at a current density of 5 kA cm^−2^, the maximum $g$ at steady state gradually increases as the TTA rate increases.

### High current injection characterization in OLEDs

According to the simulation results, the TTA process and pulse pumping with a short duration of up to 10 ns are more conducive to reaching the laser threshold. Therefore, we will investigate the device performance of α,β-ADN-based OLED devices under short electrical pulse driving. To verify the TTA characteristics of α,β-ADN: BSBDPA (5 wt%), we fabricated control devices with CBP: BSBDPA (5 wt%) emissive layers; the device performance of these two devices is shown in Fig. S5. The peak EQE of α,β-ADN: BSBDPA-based device is 6.81% which exceeds the theoretical EQE limit of ≈3% for typical fluorescent OLEDs(calculated from the PLQY of 62%, assuming singlet: triplet generation of 1:3 and light out-coupling of 20%). The peak EQE of CBP: BSBDPA-based OLED is 4% which is close to its limit 4.5%(calculated from the PLQY of 87%). At low current density, these two devices exhibited different phenomena in the luminance versus current density plots (log-log scale). Here, CBP: BSBDPA-based OLED showed characteristics typical to fluorescent OLEDs with a slope close to 1. This suggested a linear relationship between output photons and excitons generated. α,β-ADN: BSBDPA-based device showed two regimes with a slope of 1.26 below 5 mA cm^−^^2^ and a slope close to 1 above 5 mA cm^−2^.This non-linear relationship is typically suggestive of the TTA process^32^. Based on the aforementioned two phenomena, we can conclude that the device based on α,β-ADN: BSBDPA exhibits TTA characteristics. This is attributed to the high exciton utilization efficiency during the TTA process and the mitigated efficiency roll-off, resulting in an α,β-ADN: BSBDPA-based device exhibiting a significantly higher maximum luminance performance than CBP: BSBDPA-based device at the same current density.

Next, we will inject kA cm^−2^ current into the α,β-ADN: BSBDPA-based devices. Maintaining high current injection requires multifaceted thermal management on the OLED, including the use of μm-scale emitting area, nanosecond electrical pulses, heat spreaders, cryogenic cooling, and better conductive functional layers^18,36–39^. In this work, firstly fabricated micro-patterned ITO electrodes were fabricated on the 500 μm-thick Sapphire with high thermal conductivity (Fig. 1c). A 20 nm-thick ITO anode (R_sheet_≈110–150 Ω sq^−1^) enabled efficient hole injection, and then a 150-nm-thick Mo interconnect (R_sheet_≈0.3 Ω sq^−1^) was deposited to reduce series resistance. Finally, we use SiO_2_ as a PDL to separate Mo and ITO in the emitting area, preventing Mo from affecting the vertical current transmission of the device and defining the light-emitting area. Using the PDL layer, we can define a light-emitting area of any size, with a minimum achievable size of 10 × 10 μm^2^. Image under the microscope of 3 V-driven devices (Fig. S8) demonstrates an approximate 10% geometric discrepancy between fabricated and designed emission areas.

Our experimental validation of ITO/Mo substrate performance revealed critical thickness-dependent effects. The 10nm-thick ITO film exhibited discontinuous morphology, resulting in compromised J-V characteristics and reduced device brightness (Fig. S9a). Comparative analysis showed 20-nm ITO achieved J-V performance parity with 65-nm counterparts, establishing 20 nm as the minimal viable thickness for maintaining optimal electrical functionality. And ITO of this thickness exhibits an extremely high transmittance in the visible region (Fig. S6). Notably, under high voltage drive, devices with Mo as the conductor exhibit better current injection characteristics, which highlights the role of Mo (Fig. S9b). Moreover, the spatial configuration of Mo electrodes significantly influences current injection efficiency. For 20 nm-thick ITO substrates, optimal J-V characteristics were obtained when Mo circumferentially enclosed the emission zone. Increased distance between Mo and the active area elevated device resistance, resulting in degraded current injection performance (Fig. S9d). Minimizing parasitic capacitance induced by SiO₂ while maintaining low resistance was achieved by reducing the ITO/Mo/SiO₂ peripheral structure width to 10 μm, this design facilitates nanosecond-range pulse voltage operation. Thus, the finalized substrate incorporates Mo electrodes patterned to concentrically encircle the emission area.

The fabricated OLED devices with varying emission areas (3 × 3 mm² to 10 × 10 μm²) were initially characterized under direct current (DC) operation. Current tolerance testing revealed sustained operation from 1.2 A cm^−2^ to 1.1 kA cm^−2^ (Fig. S10a), with reduced thermal accumulation in smaller devices, thereby increasing the current injection that the device can withstand. Through effective suppression of singlet-heat-quenching (SHQ), the EQE roll-off was significantly alleviated (Fig. S10b), notably, the 10 × 10 μm² device maintained stable efficiency until exceeding 5 A cm^−2^, demonstrating superior device performance with high-current injection.

To achieve higher current injection, we used electrical pulses to drive the micro-light-emitting area devices described above. Due to the high capacitance of devices with areas of 50 × 50 μm^2^ and 10 × 10 μm^2^, the electrical pulses applied had a strong overshoot (Fig. S11), making it impossible to drive them using electrical pulses. Ultimately, a 15 ns electrical pulse (10 Hz) was used to drive the device with a 100 × 100 μm^2^ emitting area. Here, an ammeter is connected in series with the device to measure the current passing through it. The electrical pulse signal crossing the ammeter is shown in Fig. 3a, without a specially designed circuit, there is still an impedance mismatch between the device and the circuit, with an overshoot phenomenon present in the first 3 ns of the electrical pulse signal, this improves under high voltage conditions. At room temperature, under the drive of a 15 ns electrical pulse, the 100×100 μm^2^ device can withstand a current density of up to 13,240 A cm^−2^. This also far exceeds the predicted current density of 4 kA cm^−2^ required for net gain. Our device also exhibits excellent stability, under a voltage bias of 15 ns-100 Hz-46 V (corresponding to 4 kA cm^−2^), the device’s T_95_ is approximately 1.5 hour (Fig. S12). Under the 15 ns-electrical pulse, the maximum peak output luminance reaches up to 3.8 × 10^7 ^cd m^−2^(Fig. 3b). According to literature calculations, its peak radiant exitance is 56 W cm^−2^, which is the highest value in OLEDs^15^. The EQE versus current density characteristics of the device are shown in the Fig. 3c, where it remains greater than 4.3% at 100 A cm^−2^ and approximately 1% at 1000 A cm^−2^. Under high current density injection, the EQE of the α,β-ADN: BSBDPA-based device is about 2-3 times that of the CBP: BSBDPA-based device. Based on the extracted rate constants in Supplementary Note 1, we modeled the EQE roll-off curve. The simulated EQE was found to be in excellent agreement with the experimental results (Fig. 3c). Combined with the gain simulation (Fig. S4), we conclude that TTA and STA are two significant annihilation mechanisms that have opposite influences on the EQE roll-off. So, there are two key factors for achieving high EQE: First, utilizing short pulses can achieve a higher peak brightness, as short pulses can capture the peak signal before the roll-off of the transient EL signal and reduce the accumulation of triplets to suppress STA (Fig. 3a). Second, the TTA characteristic alleviates the STA. It decreases the triplet density and generates additional singlet excitons from triplet excitons. And according to the EL changes of the device tested under a magnetic field, the TTA characteristic remains significant at a current density of 2 kA cm^−2^ as shown in Fig. 3d. Therefore, the transient EL signal of the α,β-ADN: BSBDPA-based device shows that a peak symbolizing STA only appears when the current density exceeds 500 A cm^−2^ (Fig. S14). Utilizing short pulses allows driving the device to higher current densities before thermal breakdown, and combining TTA with short electrical pulse driving enables achieving a higher EQE. Therefore, by integrating high current injection with high EQE, we have obtained the OLED device with the highest optical output power reported thus far (Fig. 3e, f).

### Fabrication and characterization of microcavity OLED

In order to achieve laser emission, a resonator cavity is still required to provide feedback for photons. Next, we investigated the performance of micro-emitting-area devices driven at high current density within a microcavity. The bottom part consists of a DBR and an ITO layer, while the top is either a thick MgAg(1:9) metal reflector or a thin MgAg(1:9) with DBR. The central wavelength of the DBR at 496 nm, which corresponds to the ASE peak of the material. The bottom DBR was prepared by physical vapor deposition, and the top DBR was prepared by thermal evaporation. The reflectance spectrum of the reflector was simulated using COMSOL Multiphysics software. According to the simulation results, the bottom DBR consists of 13 pairs of 50.2 nm TiO_2_ and 84.3 nm SiO_2_ layers, with an additional layer of 50.2 nm TiO_2_. The simulated reflectance is close to 100%, while the actual prepared reflectance is 99.5% and the roughness is 1.65 nm. The high reflectance bandwidth and center wavelength position of the prepared sample are consistent with the simulation results. The Top DBR consists of 9 pairs of 59.9 nm-thick TeO_2_ and 91.2 nm-thick LiF layers, with an additional layer of 59.9 nm TeO_2_. The reflectance is 95%, which is consistent with the simulation results, and the roughness is 2.51 nm. Due to the smaller difference in refractive index between TeO_2_(*n* = 2.07;496 nm) and LiF(*n* = 1.36;496 nm) than TiO_2_ (*n* = 2.47;496 nm) and SiO_2_ (*n* = 1.47;496 nm), and the lower density of the film deposited by thermal evaporation compared to physical vapor deposition, the reflectance of the top DBR is lower than the bottom DBR. Moreover, high optical absorption of TeO_2_ and LiF further limited reflectivity; increasing the number of layers in the DBR will result in a higher absorption, and the reflectance of the device will not further improve. Therefore, 9.5-period TeO_2_/LiF layers were prepared to get the highest reflectance (Fig. S15). Finally, the high-reflectance bandwidth of the bottom and top DBR can encompass the PL emission spectrum of the material, reflecting the accuracy of our design and fabrication (Fig. S16e).

When coupling the light-emitting device into the resonant cavity, it is critical to fabricate electrodes on the bottom and top DBR reflectors to drive the light-emitting device. An ITO electrode is used for the bottom electrode. To reduce the negative impact of ITO on the reflectance of the DBR reflector, we deposit a 20 nm-thick thin ITO film as the electrode, as described earlier, and use Mo as the metal lead to enhance charge transport capability. Depositing a 20 nm thin film on the DBR reflector will hardly affect the reflectance of the DBR at 450-560 nm. In the 400-450 nm wavelength range, due to the strong absorption of ITO, the reflectance of the DBR + ITO mirror will decrease (Fig. S16b). A 15 nm MgAg electrode is evaporated on the top DBR. The thin MgAg electrode reduces the reflectance of the DBR by 2–3% (Fig. S16d). To verify whether the top DBR will damage the thin MgAg electrode during deposition, we also inserted an NPB as a buffer layer between top DBR and MgAg layer for comparison. It was found that the device without the buffer layer was not affected in terms of device performance. Therefore, it will be considered to directly deposit the DBR layer on the MgAg electrode (Fig. S16f).

We fabricated two distinct microcavity OLED configurations: The first architecture employed a 120-nm MgAg metal top reflector, achieving an 8.5-nm spectral linewidth at 498 nm under 13 kA cm^−2^ operation, with precise thickness tuning of functional layers aligning the emission wavelength to the cavity mode center (496 nm). Optical simulations confirmed predominant optical field localization within the emissive layer to enhance the coupling between photons and excitons (Fig. 4a, b). The second design incorporated a hybrid DBR/MgAg top reflector combined with thickened transport layers to compress the spectral linewidth to 5.5 nm while maintaining equivalent current tolerance. There are two factors jointly contributing to the narrowing of the emission linewidth: First, as shown in Fig. 4d, the top DBR exhibits higher reflectivity than the MgAg, resulting in a microcavity with a higher quality factor and lower losses. Second, according to the Schawlow–Townes theory, the relationship between microcavity length (*L*) and linewidth (∆*v*) is described by the following relationship: $\triangle v\propto {{L}^{-2}}$, where increasing the cavity length effectively compresses the linewidth. The main peak of the optical field inside the cavity is located in the emissive layer region (Fig. 4c, d). Unfortunately, the spectra of the two structural devices did not exhibit further narrowing and the “threshold” behavior of non-linear growth in output power (Fig. S17). This is attributed to the thinner gain layer’s inability to provide sufficient optical gain within the resonator cavity. Our attempt to thicken the gain layer to 120 nm to provide enough gain resulted in severely degraded I-V and EQE characteristics due to unbalanced carrier mobilities in α,β-ADN, preventing further investigation (Fig. S18).

## Conclusion

In summary, we have demonstrated OLEDs with record-high radiant exitance (56 W cm^−2^) can achieve the requirement for net gain in the α,β-ADN: BSBDPA system. And integrated OLEDs into vertical microcavities, achieving 5.5 nm spectral linewidths output. We have addressed the severe efficiency roll-off induced by STA under kA cm^−2^ current density injection in OLED, as well as the contradiction between maintaining both electrical performance and optical characteristics in device structures. We believe that further fabricating TTA-type OLED devices with optical gain characteristics, leveraging short-pulse driving, and integrating them into this high-quality microcavity structure will present an opportunity to achieve electrically pumped lasing in organic systems.

## Methods

### Materials

N,N,N’,N’-tetrakis(4-Methoxy-phenyl)benzidine (MeO-TPD;99%), 2,3,5,6-Tetrafluoro-7,7,8,8-tetracyanoquinodimethane (F4-TCNQ;99%), 9-(1-naphthyl)-10-(2-naphthyl) anthracene(α,β-ADN;99%), N,N’-(((1E,1’E)-1,4-phenylenebis(ethene-2,1-diyl))bis(4,1-phenylene))bis(2-ethyl-6-methyl-N-phenylaniline) (BSBDPA;99%), Bis(10-hydroxybenzo[h]quinolinato)beryllium(Bebq_2_;99%), Liq (99%),LiF (99%) were purchased from Volt-Amp Optoelectronics Tech. Co., Ltd. Silver pellets (Ag, 99.99%), magnesium pellets (Mg;99.99%) were purchased from Kurt J. Lesker. TeO_2_ was purchased from Macklin. The bottom DBR reflector was purchased from Wuhan Huachuang Optoelectronic Technology Co., Ltd. ITO, top DBR reflector, and the whole OLED devices were fabricated from Guangzhou New Vision Optoelectronic Technology Co., Ltd.

### Device fabrication

#### Bottom transport ITO electrode and top emitting ITO electrode fabrication

A 20nm-thick ITO electrode with low refractive index (1.81) and low sheet resistance (120 ± 15Ω per square) was sputtered via RF magnetron on the sapphire or DBR substrate. ITO was etched by the aqua regia and annealed at 600 K in N_2_. Bottom metallic contact lines were sputtered via DC magnetron over the substrates and etched by the nitric acid-based solution. A pixel definition layer (SiO_2_) was sputtered via RF magnetron on the substrates and etched by the mixed gas of CF_4_/O_2_ (20:1) to expose the active area of OLED.

The bottom electrode of the top-emitting device is ITO(10 nm)/Ag(100 nm)/ITO(10 nm), ITO and Ag were sputtered via RF magnetron on the sapphire, and etched by the aqua regia.

### Deposition of OLED functional layers

The pixel-defined substrates were cleaned in ultrasonic baths of deionized water for 10 min and baked at 450 K for 30 minutes. Organic layers and a metal electrode were then vacuum-deposited by thermal evaporation under a pressure of 1.5 × 10^−4^Pa with a total evaporation rate of 0.5-1 A s^−1^ with the structure Meo-TPD: F4-TCNQ(4%;10/175 nm)/Meo-TPD(20 nm)/CBP: BSBDPA(4%) or α,β-ADN: BSBDPA(4%)(30 nm)/Bebq_2_:Liq(2:1;30 nm)/Mg: Ag(1:9;20/120 nm).

### Top DBR reflector fabrication

The DBR was fabricated via alternating thermal evaporation deposition of TeO₂ and LiF layers at a controlled evaporation rate of 2 Å s^−1^ for both materials. A stack comprising 9.5 bilayer periods demonstrated optimal performance with a peak reflectance of approximately 95%.

### Device characterization

#### Photophysical parameter measurements

The UV-vis absorption spectra, steady PL spectra, and PLQY values were measured in a Shimadzu UV-3600 spectrophotometer, Edinburgh FLS 980 spectrophotometer, and Hamamatsu absolute PL quantum yield spectrometer C11347 Quanturus_QY, respectively. The excitation wavelengths of the PL spectrum and the PLQY measurement are 330 and 340 nm, respectively.

### ASE characterization

The third harmonic laser beam (wavelength of 343 nm, pulse width of 230 fs) of Yb:KGW laser (PHAROS, Light Conversion) was extended to a diameter to 10 mm, and then passed through a set of neutral density filters to obtain the desired energy density. A cylindrical lens and a two-dimensional slit with a micrometer were used to shape the spot as a stripe with an area of 4.3 × 1.0 mm^2^. The pump pulse energy was measured by a laser energy meter (PM100D and ES111C, Thorlabs). The spectra of the edge of the sample were collected by a fiber optic spectrometer (USB2000 + , Ocean Optics).

### VSL measurements

VSL measurements were conducted using the same configuration as in the ASE experiments, with the excited stripe-shaped area of a varied length. Fix the pump energy density (twice the threshold energy density), measure the PL spectra under different spot lengths, and use the following formula to fit the net gain coefficient g: $I={{AP}}/{g}\left[\exp \left({gl}\right)-1\right]$ *A* is constant, *P* is pump energy, *l* is stripe length. Under fixed spot size and pump energy density (greater than the threshold energy density), vary the distance (x) traveled by light within the thin film while measuring the PL spectra. The corresponding waveguide loss coefficient *a* is determined by the following formula: $I={I}_{0}\exp \left(-{ax}\right)$.

### Fs-TA and Ns-TA measurements

The Fs-TA and Ns-TA measurements were performed on the HARPIA system (Light Conversion). The pump source of Fs-TA was the third harmonic laser beam (wavelength of 343 nm, pulse width of 230 fs) of Yb: KGW laser (PHAROS, Light Conversion). White light is generated by a 515 nm femtosecond laser passing through a white-light crystal. The pump source of Ns-TA was the third harmonic frequency light with a wavelength of 343 nm generated in the HIRO module, and the white light was produced on the LEUKOS nanosecond laser at a repetition of 1800 Hz.

### D.C. driving electrical characterization

J-V characteristics of devices were obtained by a Keithley 2400 source meter unit, and the L-V characteristics were simultaneously recorded using a CS-200 chroma meter. The J-V-L characterization system was controlled via a LabVIEW program, with the sample positioned approximately 50 cm from the chroma meter. During the testing process, a 1 mm diameter optical fiber was employed to connect the QE-PRO spectrometer for spectral measurement.

### Pulsed electrical characterization

An AVIR-4-B voltage pulser (Avtech) was used to electrically pump the OLEDs through 50 Ω coaxial cables. The transient current signal was measured by an ammeter (CT6; Tektronix) on the current return. The transient emitting signal of the OLED was then collected by an amplified photodiode (Thorlabs, APD4302A). The voltage, current, and the photodiode signals were synchronously collected using the MSO44 oscilloscope. There are two methods to measure EQE: (i) the measured luminance (collected by CS-200) and calculated EQE under pulsed operation at 1 A cm^−2^ were calibrated against the measurements conducted with the DC current density of 1 A cm^−2^. This correction leads to the real luminance and EQE values of the device under high-injection currents. (ii) Place the sample into an integrating sphere(Ocean Optics) and utilize the energy correction file to correct the output energy, thereby obtaining accurate EQE values.

### MEL characterization

The sample was positioned at the center of a magnet (Standard type electromagnet-3313; East changing) capable of generating up to 300 mT, connected to the voltage pulser via 50 cm copper wires. The voltage pulser was configured with a 1000 Hz output frequency to ensure sufficient luminescence intensity. The optical signal from the sample was collected by a 20 cm focal length lens, focused onto a 1 mm diameter fiber, and transmitted to a QE PRO spectrometer for spectral analysis.

### Reflectance characterization

Absolute reflectance measurements were performed using a calibrated white reference plate (Ocean Optics) within the reflectometer to obtain the reflectance spectra. For reflectance characterization relative to an aluminum mirror, an aluminum mirror with approximately 90% reflectance was employed as the reference standard for comparative reflectivity testing.

### Radiant exitance calculation

$$\displaystyle{\mathrm{Radiant}}\,{\mathrm{exitance}}={\rm{J}}{{\eta }_{\rm{EQE}}}/{\rm{e}}\times {\rm{hc}}/{\lambda }=12.4\times {\left(\rm{J}{{\eta}_{\rm{EQE}}}\right)}/{\lambda}=56\,{\rm{W}}\,\,{\mathrm{cm}}^{-2}$$

## Supplementary information

Ultrahigh-radiSupplementary informationance TTA based OLED with 13 kA cm^−2^ Current Injection-

## Data Availability

The data that support the findings of this study are available from the corresponding authors upon reasonable request.
