# Supplementary material for: Ultrahigh-radiance TTA-based OLED with 13 kA cm−2 current injection
Source: Light Sci Appl. 2026 Jan 27;15:89. doi: 10.1038/s41377-025-02134-z (PMC12835098; doi:10.1038/s41377-025-02134-z)
Supplement: Supplementary file 1 — Ultrahigh-radiSupplementary informationance TTA based OLED with 13 kA cm−2 Current Injection- [file 41377_2025_2134_MOESM1_ESM.docx]

Supplementary Information for:

**Ultrahigh-radiance TTA based OLED with 13 kA cm^-2^ Current Injection**

Jichen Zhao^1†^, Yu Mao^1†^, Wansheng Liu^1^, Zengyi, Peng^1^, Xu Wang^1^, Jianhua Zou^1^, Jianbin Wang^1^, Dan Chen^4*^, Dongge Ma^1,3*^, Hongbin Wu^1*^, Bin Hu^1,2,3*^, Junbiao Peng^1*^

^1^ National Key Laboratory of Luminescence Materials and Devices, South China University of Technology, Guangzhou 510641, China

^2^ Spin-X Institute, South China University of Technology, Guangzhou 510641, China

^3^Guangdong Basic Research Center of Excellence for Energy and Information Polymer Materials, Guangzhou 510641, China

^4^Jihua Laboratory, Foshan, Guangdong Province, 528200, China

*Corresponding author: Junbiao Peng :[pengjb@scut.edu.cn](mailto:pengjb@scut.edu.cn) ; 13570098191; Bin Hu: [bhu@scut.edu.cn](mailto:bhu@scut.edu.cn); Hongbin Wu: hbwu@scut.edu.cn; Dongge Ma: [msdgma@scut.edu.cn](mailto:msdgma@scut.edu.cn)

Dan Chen: [wjfjs2cd@163.com](mailto:wjfjs2cd@163.com)

^†^These authors contributed equally: Jichen Zhao: [2055146951@qq.com](mailto:2055146951@qq.com) ;Yu Mao: [1301978086@qq.com](mailto:1301978086@qq.com)

mswsliu@scut.edu.cn

[pzengyi@gmail.com](mailto:pzengyi@gmail.com)

[xxWang1202@163.com](mailto:xxWang1202@163.com)

[zjh@newvision-cn.com](mailto:zjh@newvision-cn.com)

[wangjb@scut.edu.cn](mailto:wangjb@scut.edu.cn)


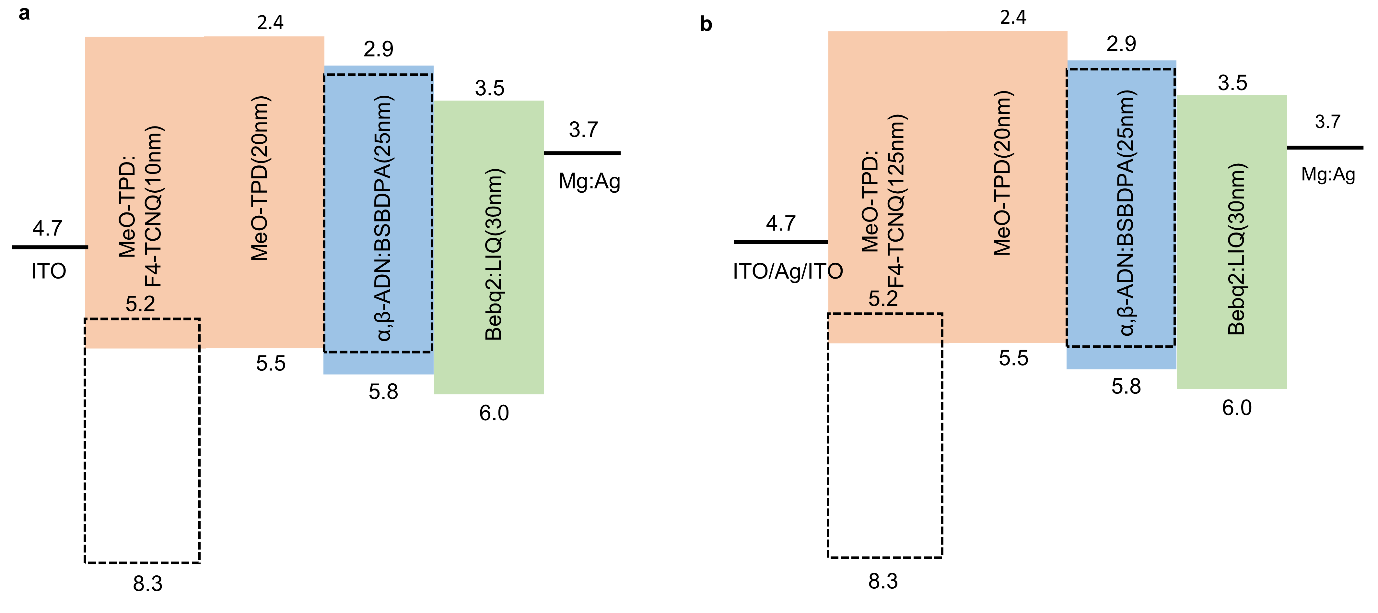


**Fig. S1.** **Energy level diagrams of bottom-emitting and top-emitting devices. a,** Bottom-emitting device. **b,** Top-emitting device.


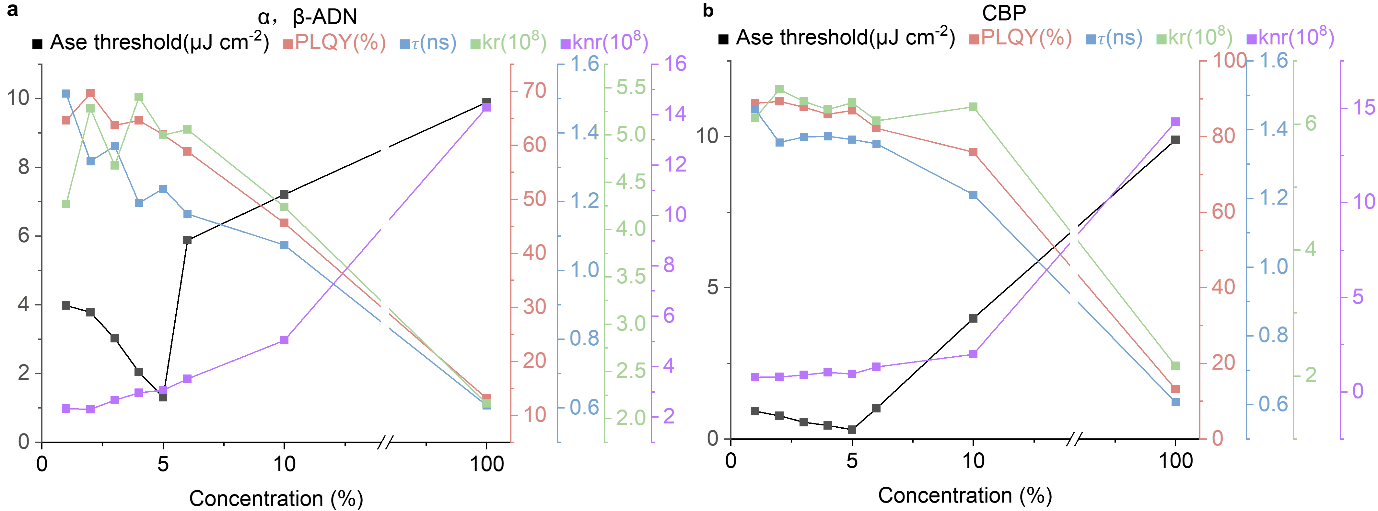


**Fig. S2. Photophysical characterization of the BSBDPA film. a,** ASE threshold, PLQY, radiative lifetime ($\tau$), radiative recombination rate (*kr*) and non-radiative recombination rate (*knr*) of 120 nm α,β-ADN:BSBDPA films with different BSBDPA doped concentration. **b,** ASE threshold, PLQY, radiative lifetime ($\tau$), radiative recombination rate (*kr*) and non-radiative recombination rate (*knr*) of 120 nm CBP:BSBDPA films with different BSBDPA doped concentration.**Supplementary Note 1. Transient Electroluminescence Analysis and Dynamic**

**Simulation** **equations Analysis :**

The transient EL at different current densities can be fitted by the following rate equations:

$\frac{dn}{dx}=\frac{J}{ed}-\gamma n^{2}$ (1)

$\frac{dS}{dt}=\frac{1}{4}\gamma n^{2}-k_{S}S$ $-k_{isc}S$ $-k_{SP}nS$ $-k_{ST}TS$ (2)

$\frac{dT}{dt}=\frac{3}{4}\gamma n^{2}-k_{T}T$ $+k_{isc}S$ $-k_{TP}nT$ (3)

$\frac{dS}{dt}=\frac{1}{4}\gamma n^{2}-k_{S}S$ $-k_{isc}S$ $-k_{SP}nS$ $-k_{ST}TS+$ $\frac{1}{4}k_{TT}T^{2}$ (4)

$\frac{dT}{dt}=\frac{3}{4}\gamma n^{2}-k_{T}T$ $+k_{isc}S$ $-k_{TP}nT$ $-$ $\frac{5}{4}k_{TT}T^{2}$ (5)

Where e, d, J, n, S, T represent the elementary electron, recombination width of excitons, current density, polaron population, singlet population, and triplet population, respectively, *kr, knr, k_T_, kisc, k_TT_, k_ST_, k_SP_*, and *k_TP_* are the rate constants of singlet decay radiative and nonradiative decay, triplet decay, intersystem crossing (ISC), TTA, STA, SPA, and TPA, respectively. γ is the Langevin recombination rate, which is γ is related to the polaron mobility μ_h_, μ_e_, i.e. $\gamma=\frac{e}{\varepsilon_{1}\varepsilon_{0}}\left( \mu_{h}+\mu_{e} \right)$.

Equation (1) describes the polaron changes. Each neutral polaron pair can form an exciton and a neutral molecule by Langevin recombination, which drives the current and leads to the reduction of polarons in equation (1).

Equation (2) describes the change in singlet density of CBP doped devices. The first term on the right-hand side (r.h.s.) is the singlet exciton produced by the polaron recombination described above. The factor 1/4 comes from the spin selection law of random injection. The second term describes the decay of singlet excitons due to radiative and non-radiative processes. The third term describes the decay of singlet excitons due to the intersystem crossing (ISC) process, with a decay rate of *k_ISC_*. The last two decay terms describe the reduction of the singlet density by the bimolecular annihilation processes of singlet-polaron annihilation (SPA) and singlet-triplet annihilation (STA), with decay rates of *k_SP_* and *k_ST_*, respectively. In the α,β-ADN doped devices with the TTA effect (As Eq. 4), there is also a generation term of singlet excitons generated by triplet-triplet annihilation, with a generation rate of *k_TT_*.

Equation (3) describes the change in triplet density of CBP doped devices. The first-generation term is the contribution from polaron recombination with a factor of 3/4 due to the spin statistics. This term plus the first singlet generation term in Equation (2) corresponds to the reduction term in Eq. 1) due to polaron recombination. The second term corresponds to the decay of triplet excitons at a *k_T_* rate. The third term describes the increase in triplet states due to ISC in the same way as the decrease in singlet states in Eq. (2). The fourth term corresponds to triplet polaron annihilation (TPA). In α,β-ADN doped device with TTA (Eq. 5) there is also a decrease in triplet density due to triplet-triplet annihilation (TTA).

**Parameter extraction:**

As shown in **Note Figure 1**, it can be seen that after the electrical pulse is turned off, there is a clear delayed luminescence in the device, and at lower current densities, the delay ratio increases with increasing current density, after which this delay ratio reaches a saturation value; in addition, the decay curve of the delayed luminescence at lower current densities is basically in the form of a mono-exponential decay, which is similar to the previously reported TTA behavior. We can extract the TTA rate of α,β-ADN doped devices as 2.15×10^-13^ cm^3^ s^-1^ by fitting the slope of the delay ratio with increasing current density according to equation $R=\frac{kTT\left( 1-\alpha\right)2}{2edhk_{T}^{2}\alpha}J$^.^

^
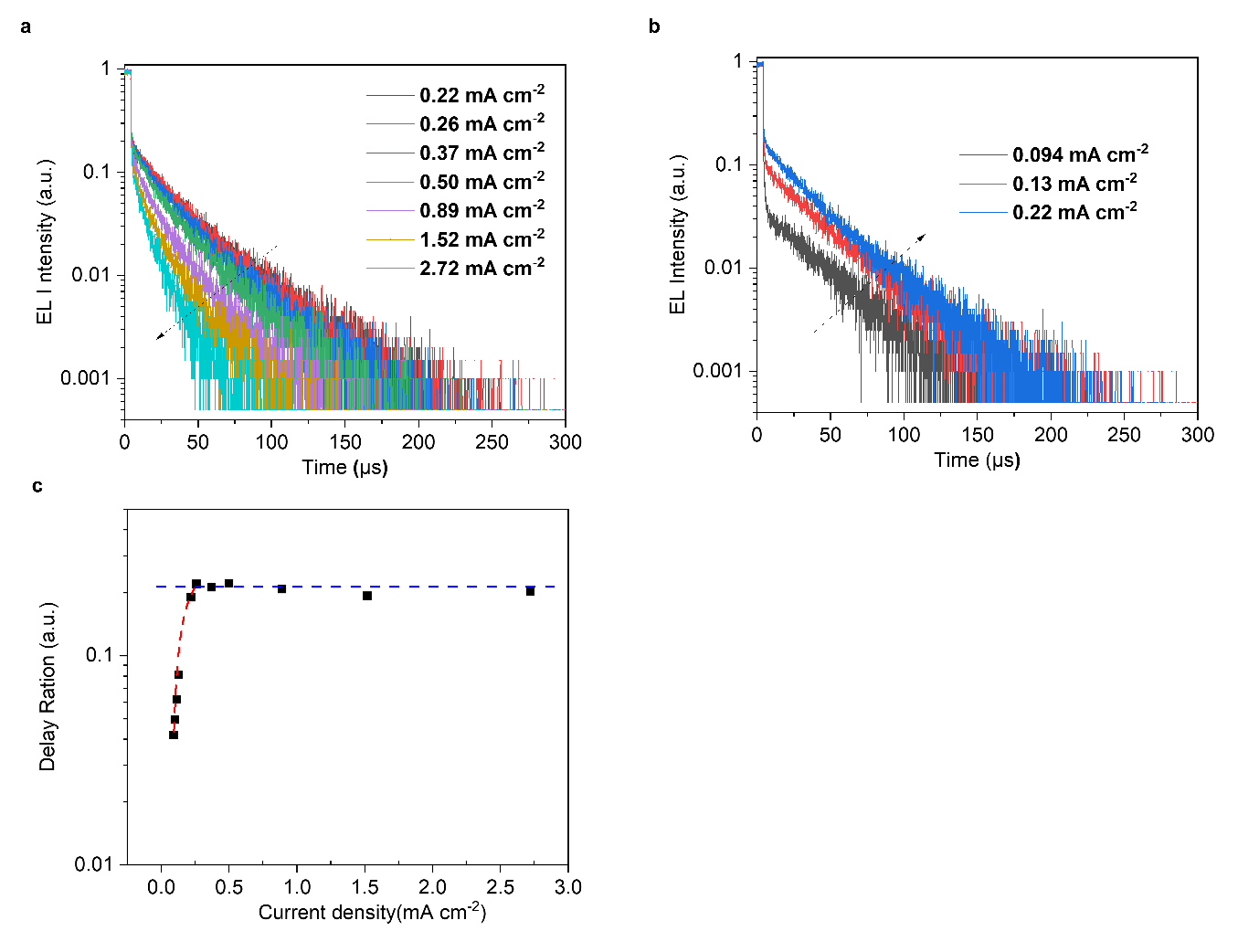
^

**Note Fig. 1. The delayed fluorescence of 𝛼,𝛽-ADN:BSBDPA based devices at varies current density.** The delayed fluorescence after the turn-off of different current pulses. **a,** EL intensity versus time which current density above 0.22 mA cm^-2^. **b,** EL intensity versus time which current density below 0.22 mA cm^-2^, **c,** TTA delay ration versus current density.

As shown in Note Figure 2, by choosing reasonable initial values for the fitted rate constants, we can fit the decay curves well at different current densities at the same time and obtain accurate and reliable rate constants. Looking at the TrEL attenuation curves at different current densities, we can see that the attenuation curves of the CBP doped devices show an increasingly pronounced peak as the current density increases. This is due to the presence of the singlet-triplet annihilation STA process in the system, which leads to a decrease in luminescence intensity compared to the initial value after reaching steady state. An overshoot ratio (OR) can be defined as the spike intensity divided by the steady-state intensity. The presence or absence of the spike, or the overshoot ratio OR, is usually used to initially determine the severity of the STA quenching process. As the current density increases, the OR of the CBP doped vice gradually increases to 1.18, indicating that the STA has a certain negative effect in this system. For the α,β-ADN doped devices, the overshoot phenomenon is very insignificant, indicating that the STA is very weak in this system.

The initial and final rate values of the fitting are given in Note table 1 and Note table 2.


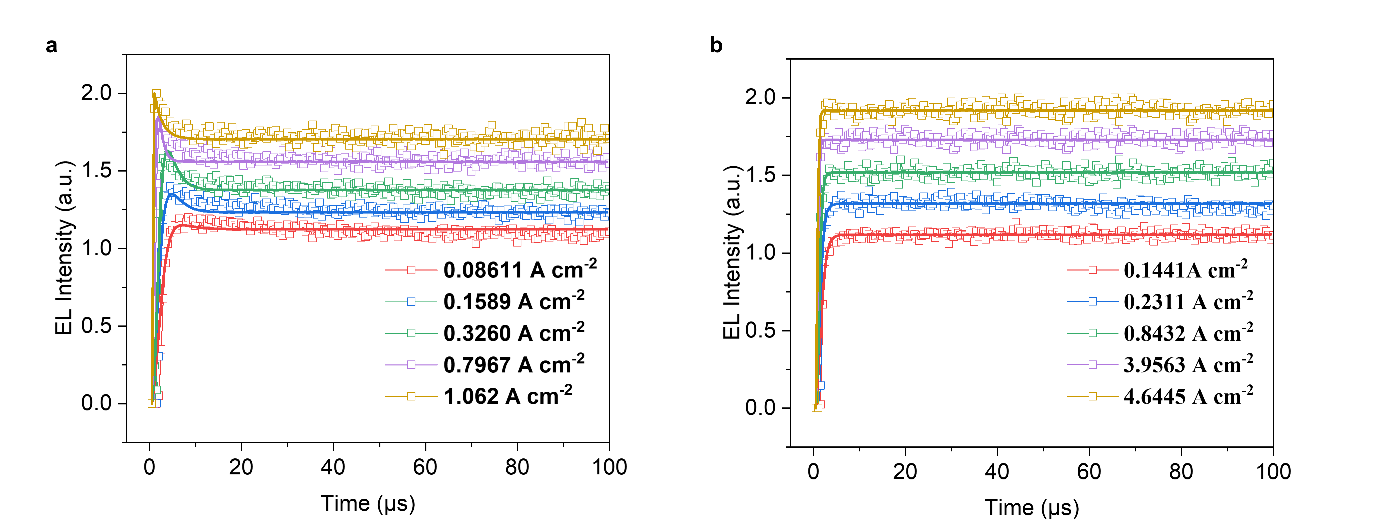


**Note Fig. 2. The transient EL of CBP:BSBDPA and 𝛼,𝛽-ADN:BSBDPA based devices at varies current density. a,** CBP:BSBDPA based OELD. **b,** 𝛼,𝛽-ADN:BSBDPA based OLED.

**Note table 1 | Experimental and fitted decay rates parameters**

|  | **experimental value** | | **fitted value** | |
| --- | --- | --- | --- | --- |
| decay rates [s^-1^ ] | CBP:BSBDPA | 𝛼,𝛽-ADN:  BSBDPA | CBP: BSBDPA | 𝛼,𝛽-ADN:  BSBDPA |
| singlet decay rate (k_S_) | 7.3×10^8^ | 7.6×10^8^ | 6.5×10^8^ | 7.8×10^8^ |
| intersystem crossing (k_ISC_) | 6.0×10^6^ | | 1.0×10^7^ | 9.3×10^6^ |
| triplet decay rates (k_T_) | 5.9×10^6^ | | 5.0×10^6^ | 5.6×10^6^ |

**Note table 2 | Initial and fitted annihilation rates parameters**

|  | **initial value** | | **fitted value** | |
| --- | --- | --- | --- | --- |
| annihilation rates [cm^3^s^-1^] | CBP: BSBDPA | 𝛼,𝛽-ADN:  BSBDPA | CBP: BSBDPA | 𝛼,𝛽-ADN:  BSBDPA |
| singlet-triplet (k_ST_) | 1.9×10^-10^ | | 2.0×10^-10^ | 1.0×10^-10^ |
| triplet-triplet (k_TT_) | 0 | 2.2×10^-13^ | 0 | 8.7×10^-14^ |
| singlet-polaron (k_SP_) | 3.0×10^-13^ | | 2.3×10^-14^ | 4.3×10^-13^ |
| triplet-polaron (k_TP_) | 2.8×10^-13^ | | 2.78×10^-13^ | 2.2×10^-14^ |
| total mobility [cm^2^ V^-1^ s^-1^ ] | 1.0×10^-6^ | 1.0×10^-6^ | 5.0×10^-7^ | 9.1×10^-6^ |

**Electrical gain simulation**

We then simulate the gain characteristics of the doped devices under electrical injection. Solving the dynamic equations gives the variation of the triplet density and singlet density with time as well as the current density, which is then introduced into the net gain coefficient function $G=\Gamma{（\sigma}_{em}S-\sigma_{TT}T)-\alpha_{cav}$, and the gain coefficient curve with time as well as the current density can be calculated. Thus, we could get the current density required to produce net gain. Stimulated emission cross-section $\sigma_{em}=\frac{\lambda^{4}f(\lambda)\Phi_{PL}}{8\pi n^{2}c_{0}\tau_{s}}$and triplet absorption cross-section $\sigma_{TT}=\frac{1000ln10\varepsilon_{TT}(\lambda)}{N_{A}}$ distributions are shown in Note Figure 3. Synthesizing previous literature reports, we can take the optical loss factor 𝛼_𝑐𝑎𝑣_ to be 80 cm^-1^ and the waveguide limiting factor 𝛤 to be 0.7.

**
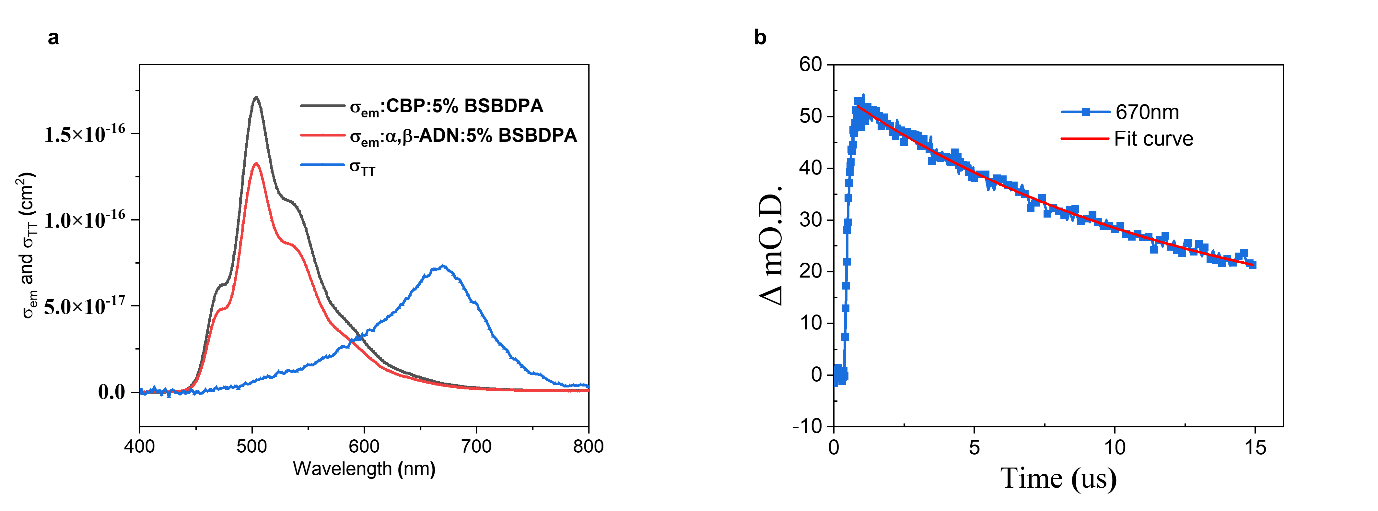
**

**Note Fig. 3. Stimulated emission cross-section and triplet absorption cross-section of BSBDPA and the dynamic process of triplet absorption. a,** $\sigma_{em}$ of CBP:BSBDPA and 𝛼,𝛽-ADN:BSBDPA, $\sigma_{TT}$ of BSBDPA. **b,** The dynamic process of triplet absorption at 670 nm.


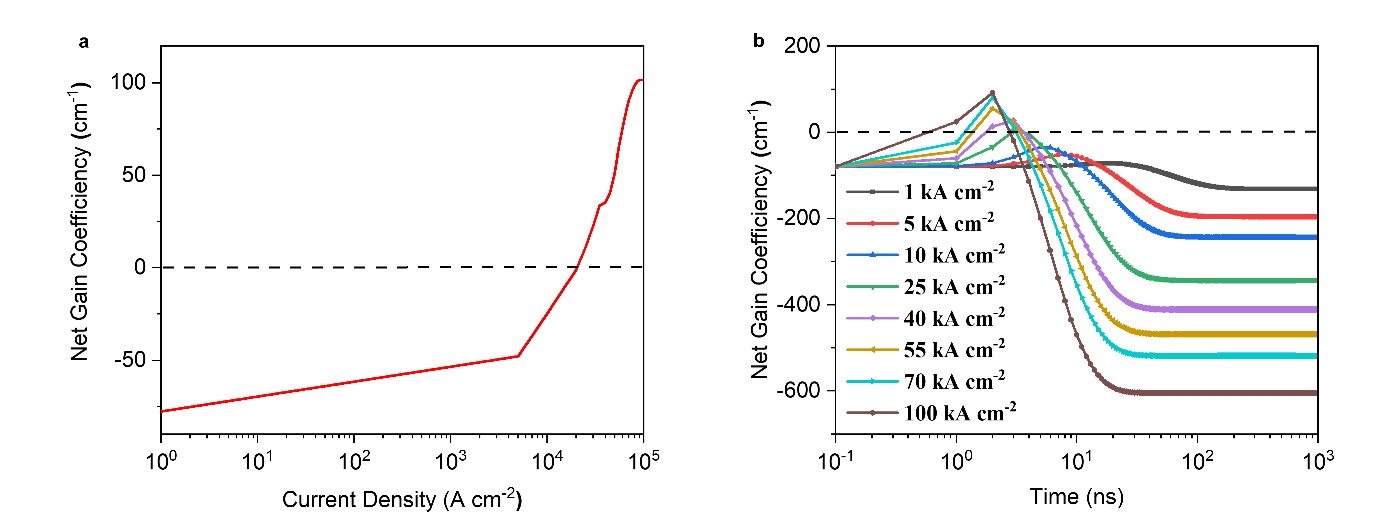


**Note Fig. 4. Gain simulaion of CBP:BSBDPA based OLED. a,** Net gain versus current density. When current density above 23 kA cm^-2^, it present positive net gain. **b,** Net gain versus time at different current density.


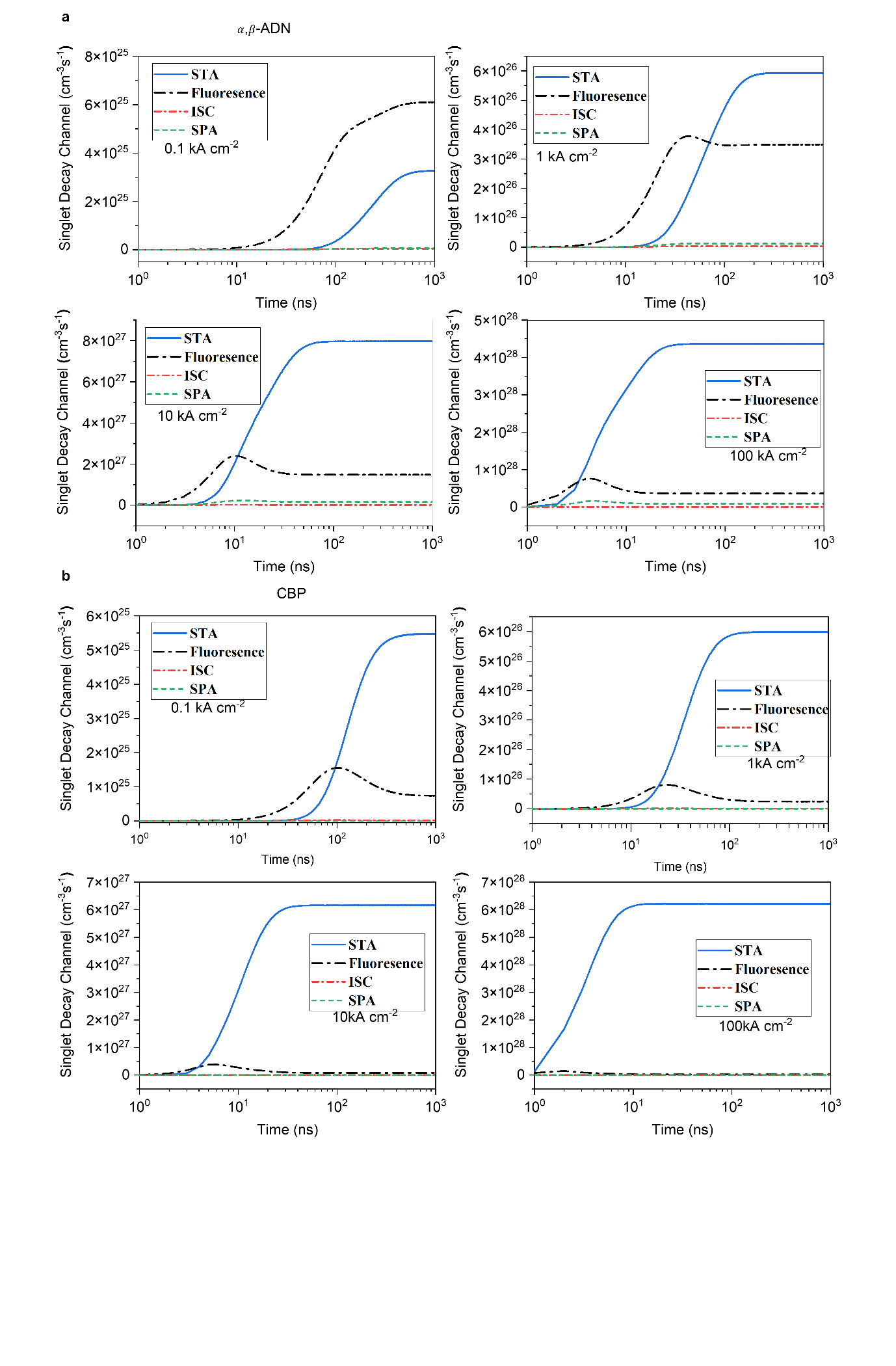
**Fig. S3. Simulation of exciton annelation process. a,** Dynamic evolution process of fluorescence, STA, ISC, SPA in α,β-ADN:BSBDPA based OLED. **b,** Dynamic evolution process of fluorescence, STA, ISC, SPA in CBP:BSBDPA based OLED.


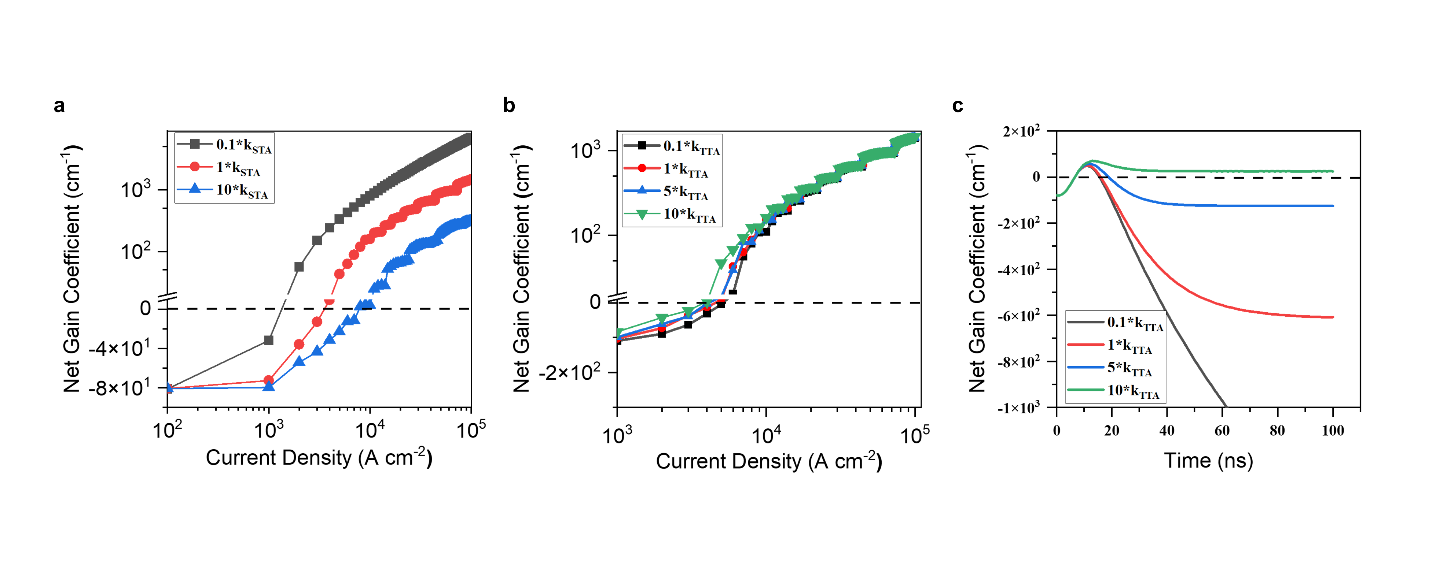
 **Fig. S4. Simulation of of key factors affecting gain. a,** Net gain versus current density with different k_STA_. **b,** Net gain versus current density with different k_TTA_. **c,** Net gain versus time with different k_TTA_ within 5 kA cm^-2^ current injection.


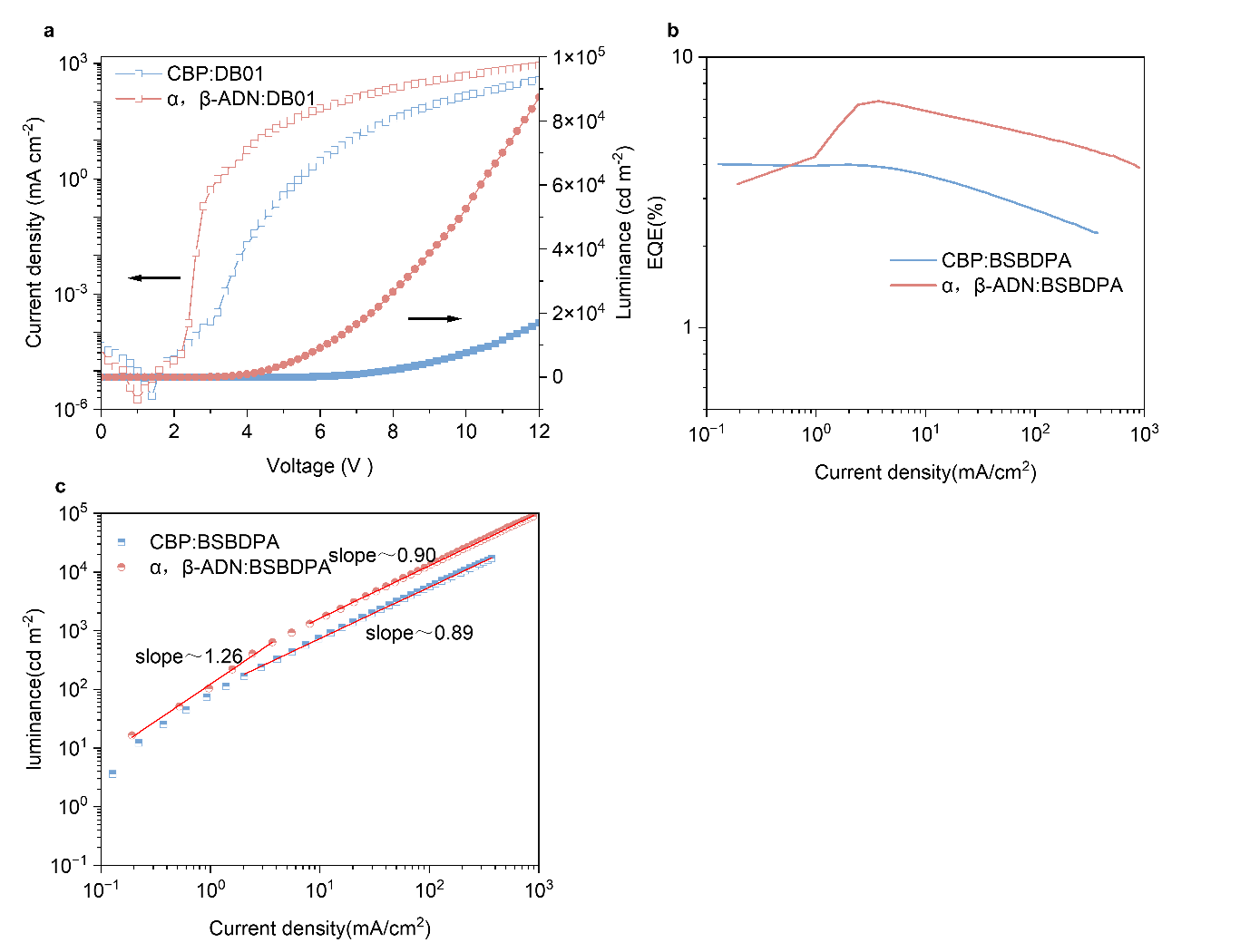


**Fig. S5. Performance comparison of α,β-ADN:BSBDPA and CBP based OLEDs a,** I-V-L characterization. **b,** EQE roll off characterization. **c,** Luminance as a function of current density.
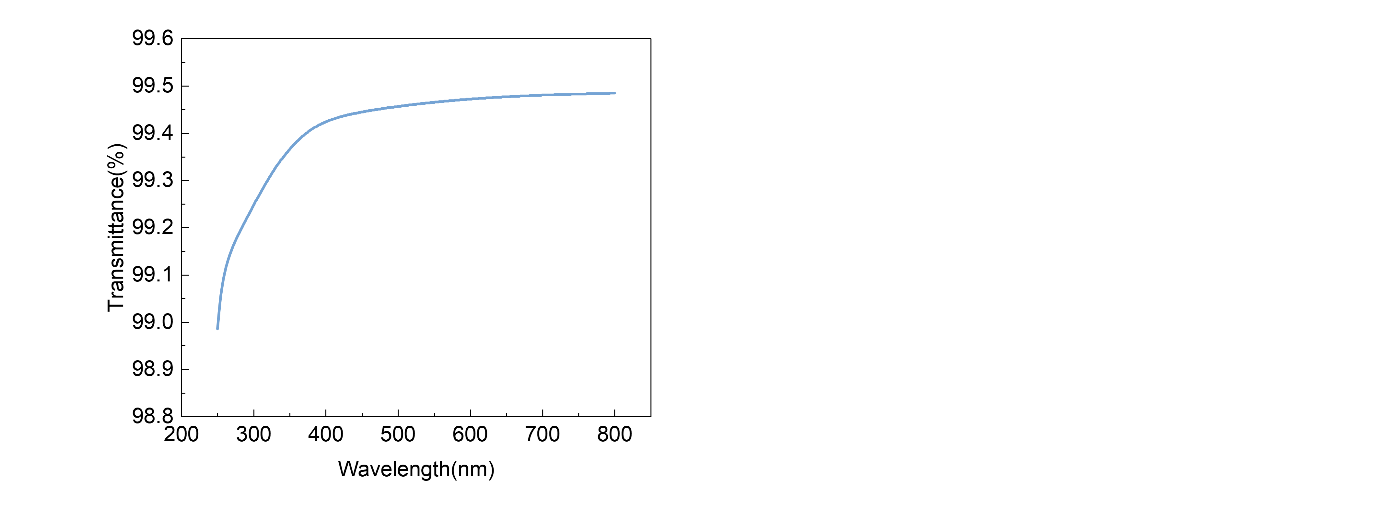


**Fig. S6. Transmission spectrum of the 20 nm-thick ITO monolayer**


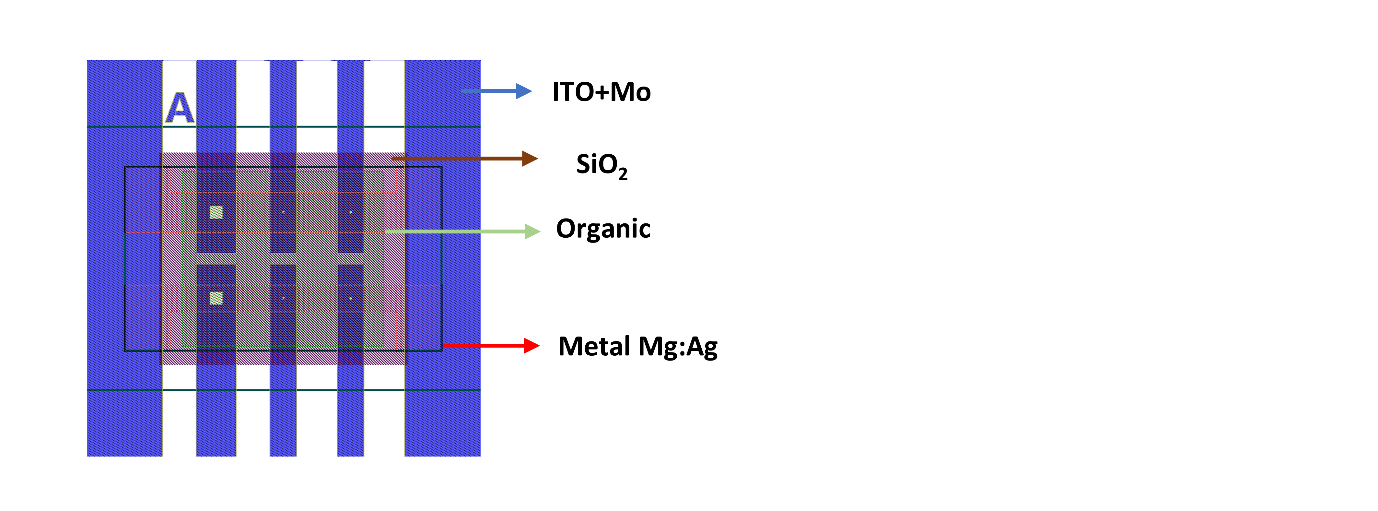


**Fig. S7. The top view structure of the OLED device.**


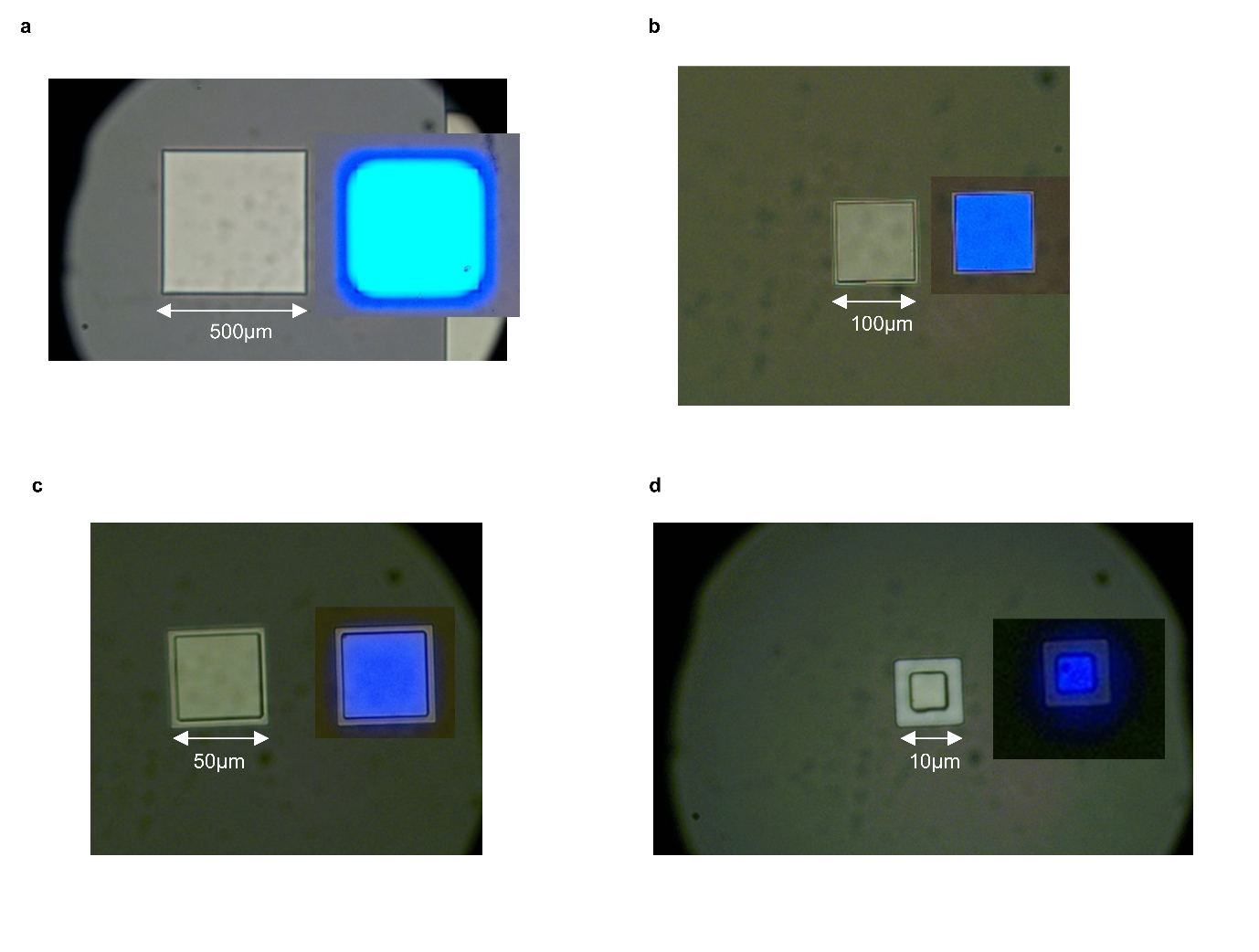


**Fig. S8. Microscopy Images of the Light-Emitting Area in the ON and OFF States.** There are four different light-emitting area. **a,** 500×500 μm^-2^.**b,** 100×100 μm^-2^. **c,** 50×50 μm^-2^. **b,** 10×10 μm^-2^.


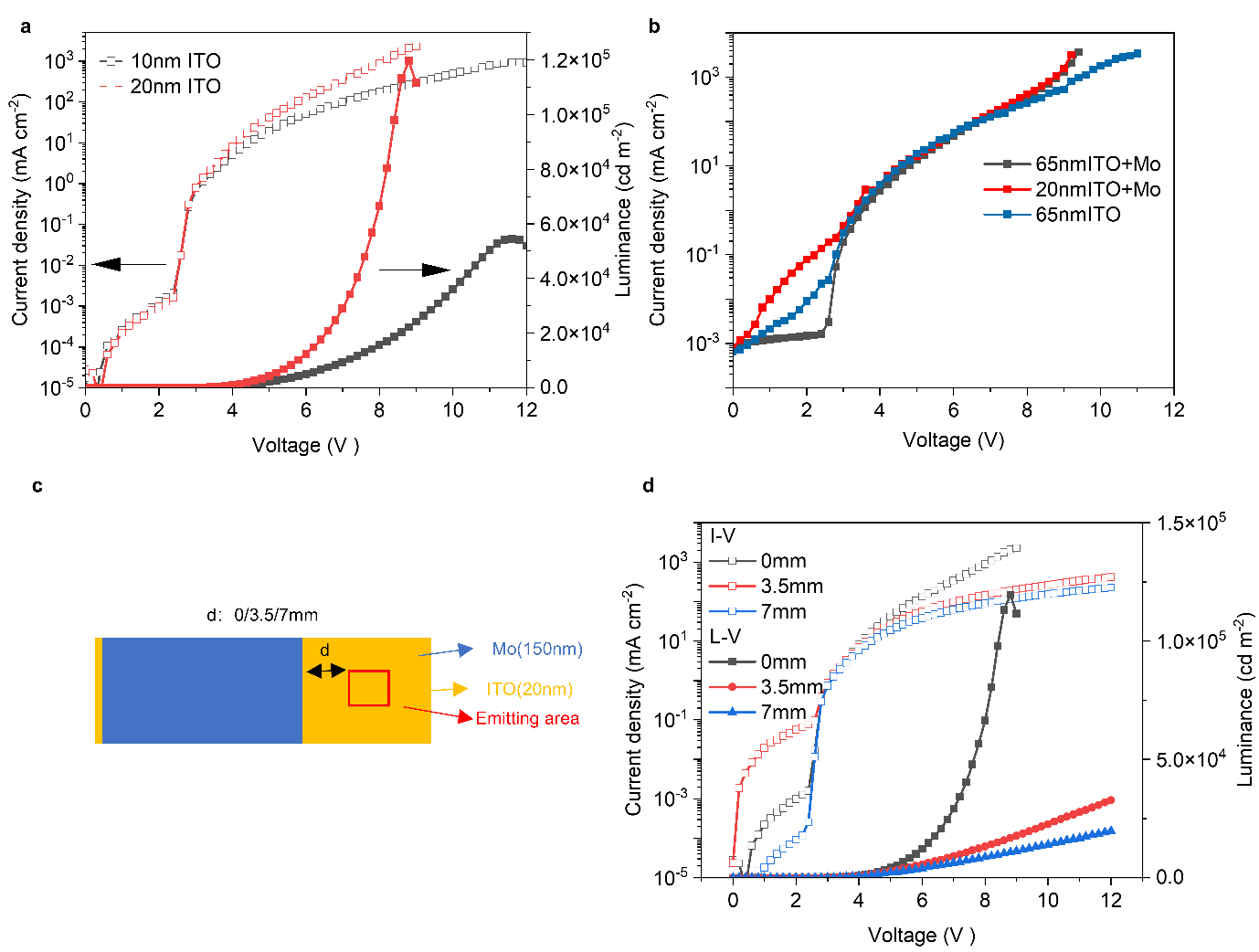


**Fig. S9. Characterization of 𝛼,𝛽-ADN:BSBDPA based OLED with different ITO and Mo structure. a,** I-V-L properties with different ITO thickness of ITO/Mo electrode. **b,** I-V properties with different ITO and Mo combination. **c,** Schematic illustrations of Mo at different distances from the light-emitting region. **d,** I-V-L properties with different distance between Mo and emitting area.


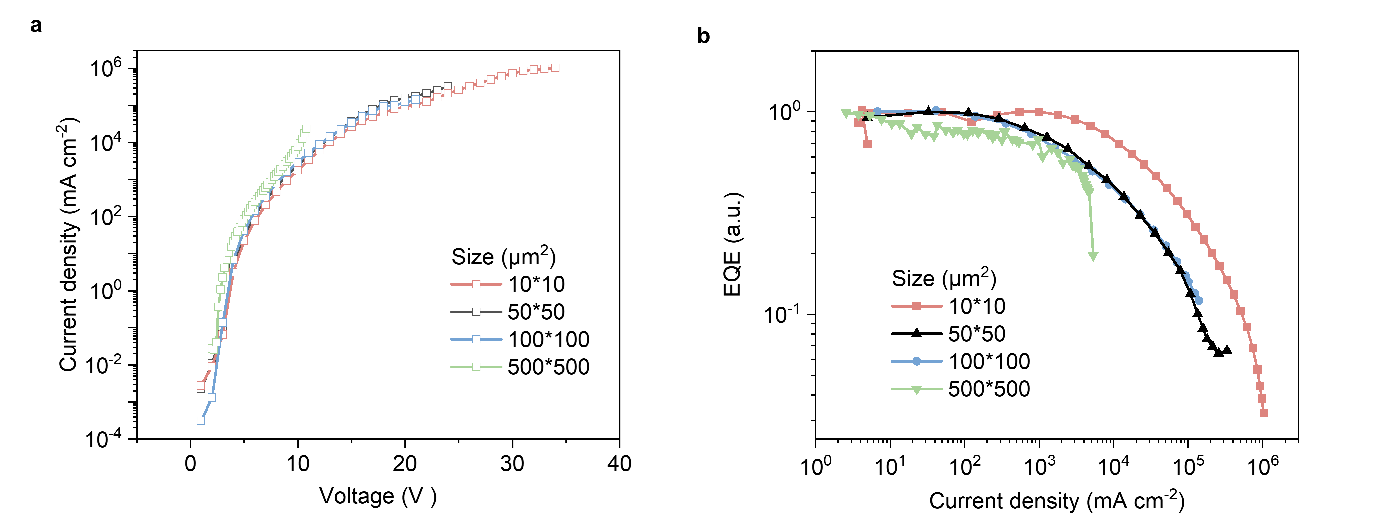


**Fig. S10. μm^2^-emitting area OLED characterization under D.C. operation. a,** I-V characterization. **b,** EQE roll off characterization.


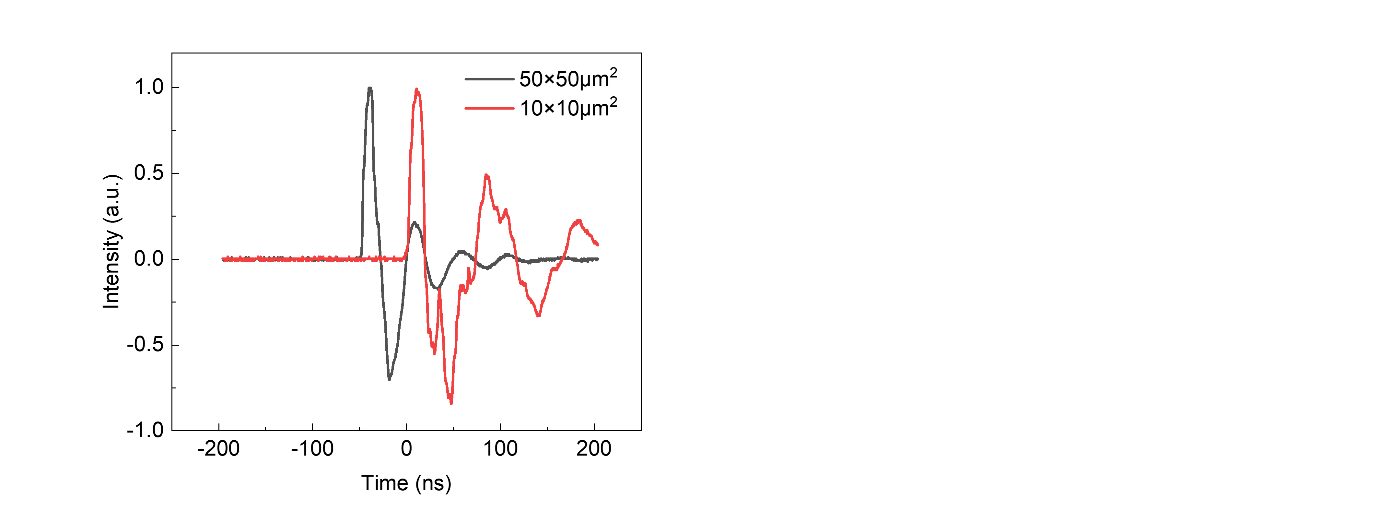


**Fig. S11. Transient current signals from devices with 10×10 μm^2^ and 50×50 μm^2^emissive areas under 15 ns electrical pulse.**


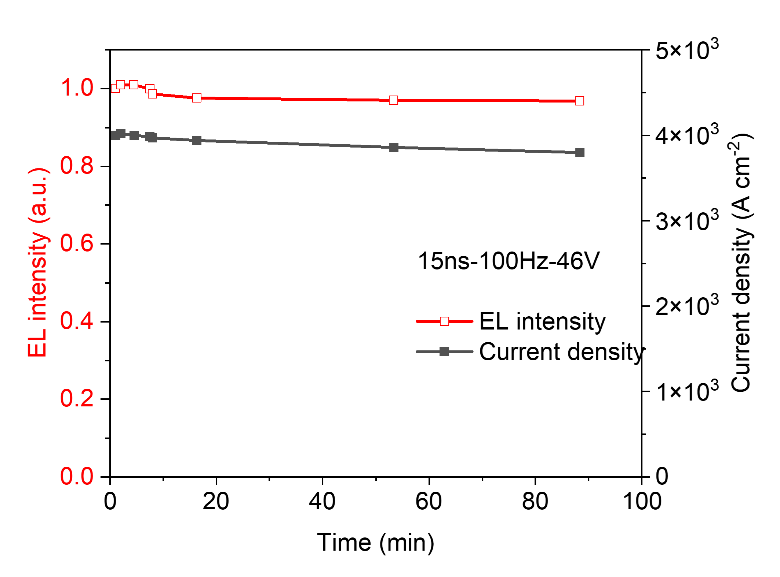


**Fig. S12.** **Lifetime of pulse-driven OLEDs.** α,β-ADN:BSBDPA based OLED driven by 15 ns-100 Hz-46 V electrical pulse. T_95_ is nearly 1.5 h.


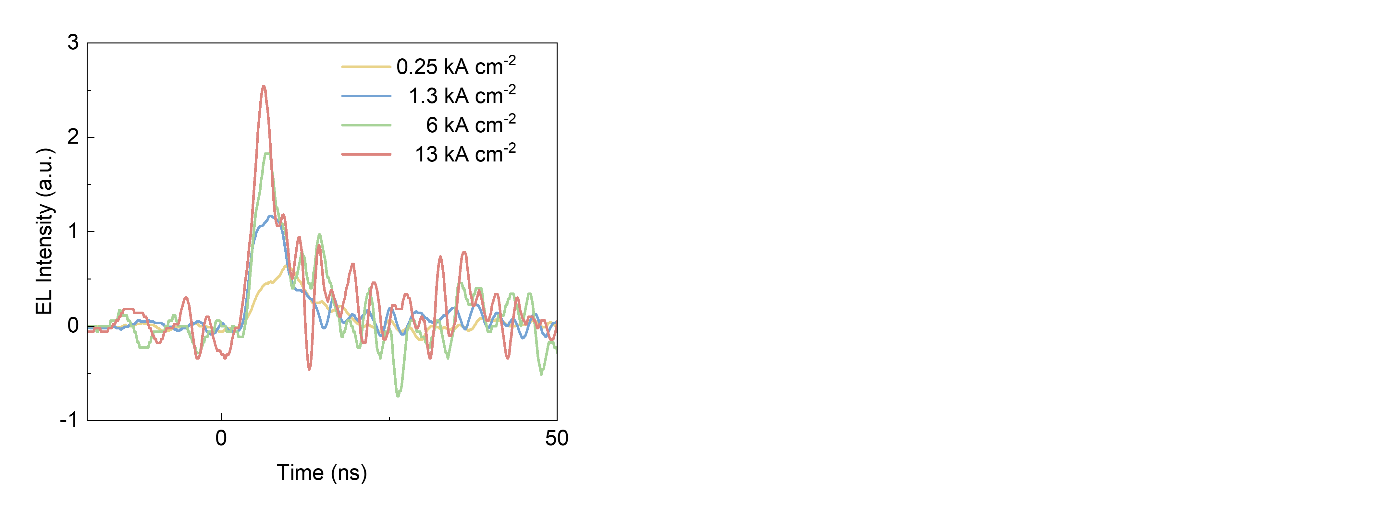


**Fig. S13. Transient EL signal of the device driven by 15 ns electrical pulses.**


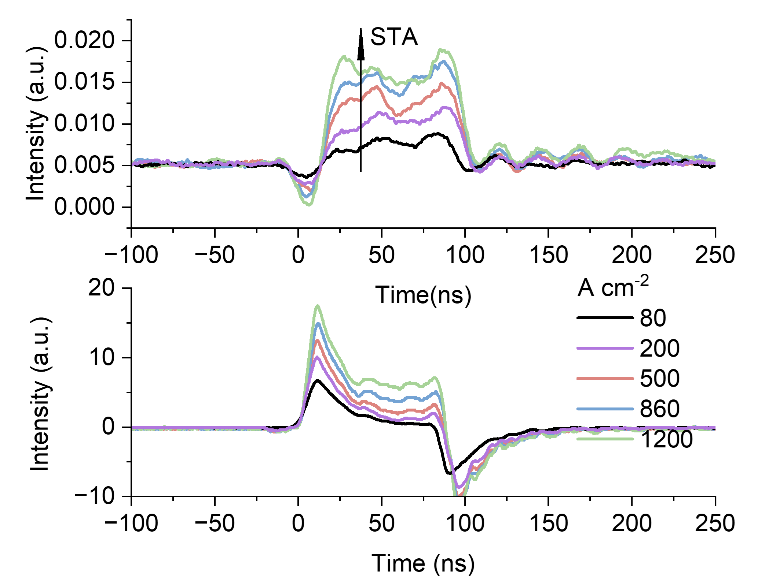


**Fig. S14.** **Transient current and EL signal of α,β-ADN:BSBDPA based OLEDs.** α,β-ADN:BSBDPA based OLED driven by 80 ns-10 Hz electrical pulse. Overshoot of STA appears above 500 A cm^-2^.


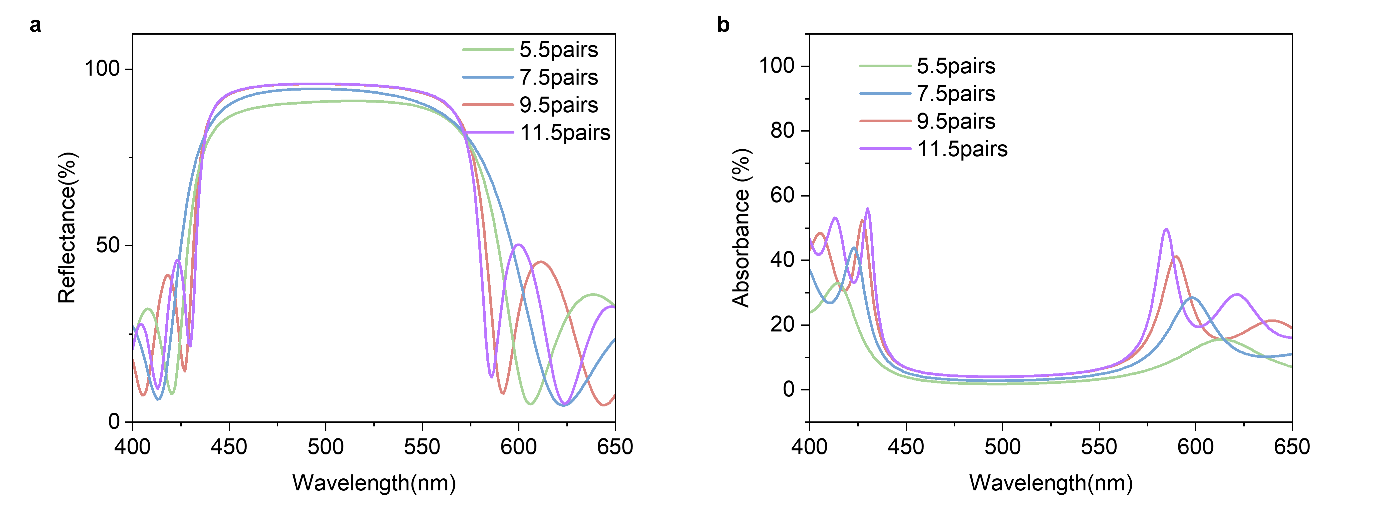


**Fig. S15. Characterization of top DBR with different layers. a,** Reflection spectrum with different pairs [TeO_2_(59.9 nm)/LIF(91.2 nm)]. **b,** Absorption spectrum with different pairs [TeO_2_(59.9 nm)/LIF(91.2 nm)].**Supplementary Note 2: Optical waveguide simulation:**

In order to simulate the optical modes of the waveguide structure of the complete device, the finite element method (FEM), implemented in the fluctuating optics module of the COMSOL Multiphysics software, was employed. The waveguide parameters were defined in accordance with the device structure that had been the subject of experimental study. The waveguide model is two-dimensional (X-Y) and was constructed based on the multilayer structure The refractive indices (n) and extinction coefficients (k) are either measured experimentally or imported from the COMSOL material library. The extinction coefficients of the remaining layers, with the exception of Ag, were not considered. Here, we consider only the transverse electric (TE) mode, which is calculated by solving the following frequency-domain wave equation.

$\nabla\times\left（ \nabla\times\mathbf{E} \right）-k_{0}^{2}\varepsilon_{r}\mathbf{E}=0$ (6)

where $\varepsilon_{r}$ (relative permittivity) was computed from $\varepsilon_{r}={(n-ik)}^{2}$. In order to solve this equation, we apply ‘scattering-type’ boundary conditions at the top air interface and the bottom glass layer. A ‘perfect’ electrical conductor boundary condition is used on the left and right sides of the model, and in order to simulate the waveguide modes, the left boundary of the waveguide structure is set to a ‘user-defined’ port, and the polarisation of the electrical mode field is set to the Z-direction. The spatial distribution of the electric field (**E**) of a wave propagating from the left edge of the waveguide structure has been determined.


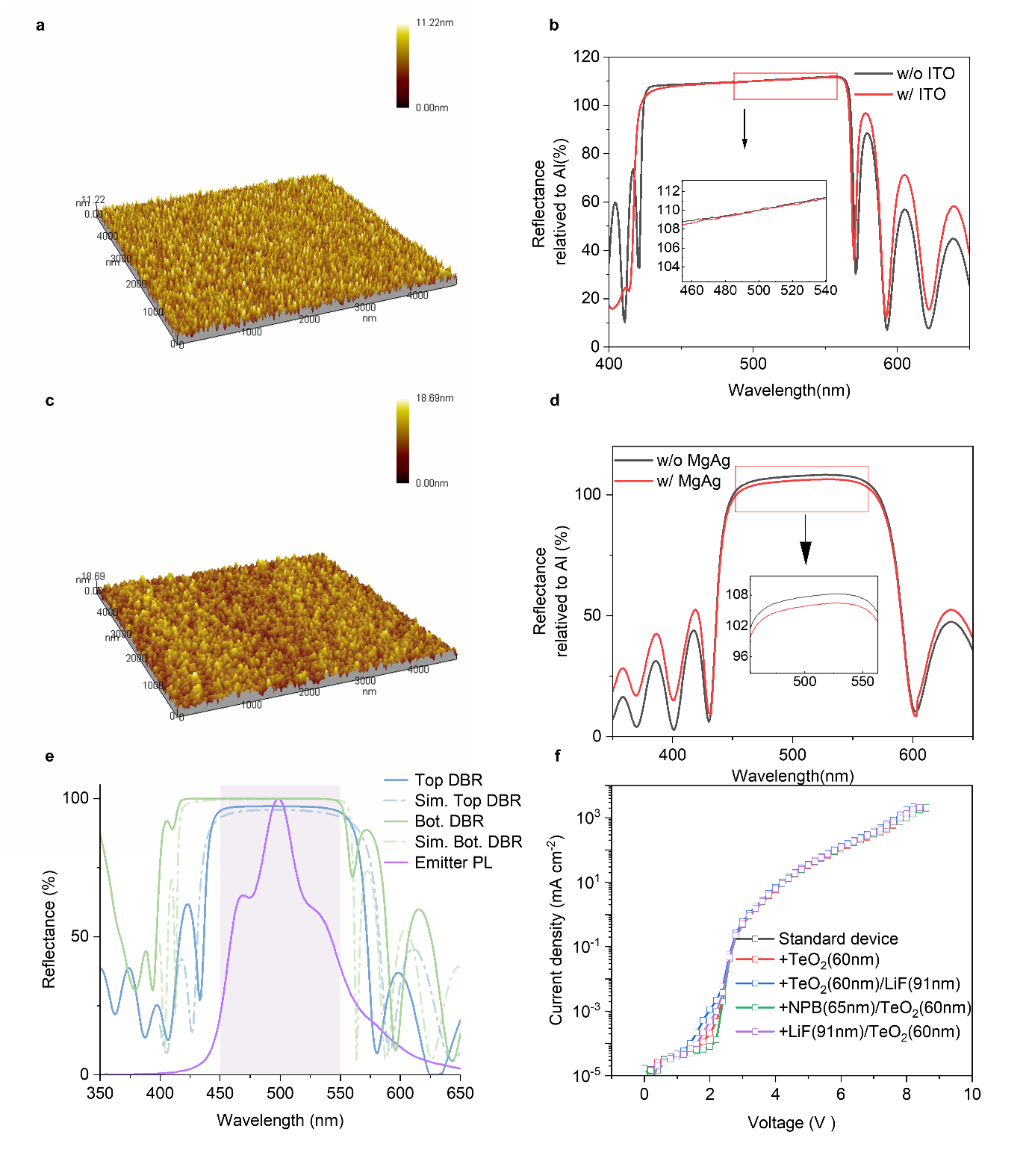


**Fig. S16. Performance of bottom and top DBR a,** AFM image of bottom DBR, the roughness is 1.65 nm. **b,** Comparison of reflectance spectra for bottom DBR with and without a 20-nm-thick ITO layer. **c,** AFM image of top DBR, the roughness is 2.51 nm. **d,** Comparison of reflectance spectra for top DBR with and without a 15-nm-thick MgAg(1:9) layer. **e,** Comparison of simulated and experimental reflectance spectra for bottom and top DBRs**. f,** I-V characterization of DBR deposited on OLEDs.

**
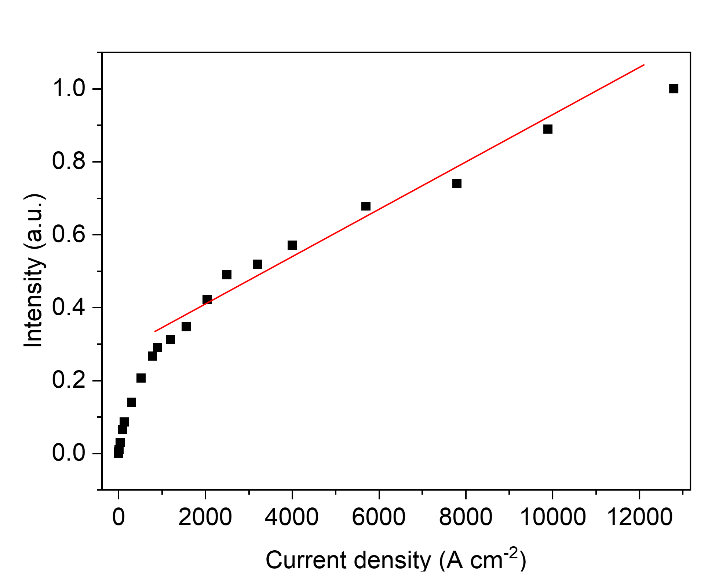
**

**Fig. S17. Output intensity of the microcavity OLED.** Output intensity of the DBR-DBR OLED versus current density, presents a linear ship between intensity and current density.


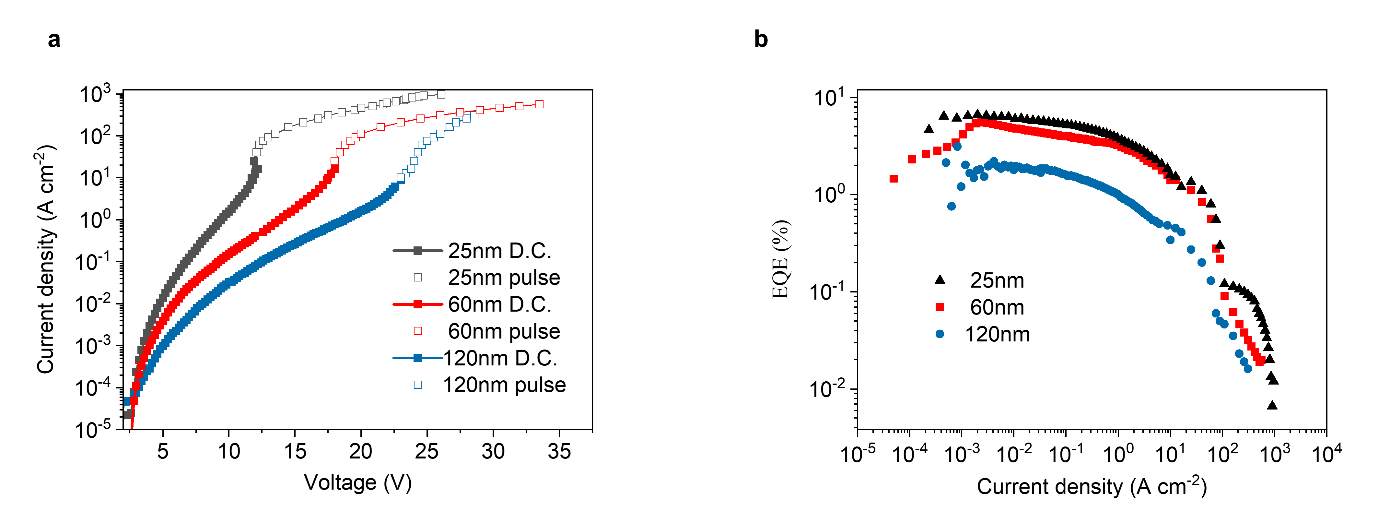


**Fig. S18. I-V and EQE characterization with different emitting layer thickness.** Driven by 1 μs electrical pulse **a,** I-V characterization **b,** EQE characterization.
